# ﻿The distribution and evolution of muscarine and the ibotenic acid biosynthetic gene cluster within the genus *Amanita* section *Amanita* revealed by phylogenomics

**DOI:** 10.3897/imafungus.17.175874

**Published:** 2026-01-09

**Authors:** Yu-Ting Su, Zheng-Mi He, Yu-Zhi Yang, Fei Xu, Meng-Meng Lai, Zhu L. Yang, Ping Zhang, Zuo-Hong Chen

**Affiliations:** 1 College of Life Sciences, Hunan Normal University, Changsha 410081, China Hunan Normal University Changsha China; 2 School of Life Science and Resources and Environment, Yichun University, Yichun 336000, China Yichun University Yichun China; 3 Ningxia Center for Disease Control and Prevention, Yinchuan 750004, China Ningxia Center for Disease Control and Prevention Yinchuan China; 4 CAS Key Laboratory for Plant Diversity and Biogeography of East Asia, Kunming Institute of Botany, Chinese Academy of Sciences, Kunming 650201, China Kunming Institute of Botany, Chinese Academy of Sciences Kunming China

**Keywords:** *
Amanita
*, ibotenic acid, muscarine, muscimol, phylogenomic analysis, single-copy gene

## Abstract

Numerous severe cases of neurotoxic mushroom poisoning worldwide are caused by ibotenic acid and muscimol produced by specific species belonging to section Amanita of the genus *Amanita*. Recent studies have demonstrated that both toxins are produced through the ibotenic acid biosynthetic gene cluster (*ibo*BGC) in these species. In addition to these two toxins, section Amanita is also thought to include several species producing another neurotoxic compound, muscarine. However, the taxonomic distribution and evolutionary history of these toxins within the section remain poorly understood. In this study, phylogenetic analyses based on nucleotide sequences of two loci (ITS and LSU) and five loci (ITS, LSU, *RPB2*, *TEF1*, and *TUB2*), together with a phylogenomic analysis using 467 single-copy genes, were conducted to reconstruct the phylogenetic framework of section Amanita. BEAST analysis was used to estimate divergence times within the section. Gene identification of the *ibo*BGC was conducted using 25 *Amanita* genomes, followed by phylogenetic analyses of each *ibo* gene. Biochemical analysis of muscarine was performed on 31 representative species. Based on these analyses, *ibo* genes were detected in 21 species forming a major monophyletic clade within the section Amanita, whereas muscarine was detected in eight species that constituted a small subclade nested within this clade. Finally, our phylogenetic, phylogenomic, chemotaxonomic, and molecular dating results indicate a monophyletic distribution of the *ibo*BGC and muscarine within the section Amanita, with independent origins approximately 28 million years ago (Mya) and 15 Mya, respectively, and no evidence of subsequent losses.

## ﻿Introduction

*Amanita*, which encompasses approximately 650 species, is the type genus and the largest genus within the family *Amanitaceae* (*Agaricales*, *Basidiomycota*) (Cui et al. 2018). The genus is well known for the significant danger posed by the basidiocarps of its numerous species, which produce a variety of toxins (Chen et al. 2014, 2016). Taxonomically, *Amanita* is classified into three subgenera, viz., subgen. Amanita, *Amanitina*, and *Lepidella*, which are further subdivided into 11 sections (Cui et al. 2018). Poisonous *Amanita* species from different sections can synthesize different types of toxins, leading to varied clinical symptoms when ingested by humans. Most species of sect. Phalloideae (e.g., *A. phalloides*) induce hepatotoxicity due to amatoxins (e.g., α-amanitin) (Cai et al. 2014; Tang et al. 2016; Diaz 2018). Some species from sects. Amidella and Roanokenses (e.g., *A. proxima* and *A. smithiana*) are known to cause nephrotoxicity attributed to aminohexadienoic acid (Diaz 2005; Kirchmair et al. 2012; Fu et al. 2017). In contrast to cytotoxic species, certain members belonging to sect. Amanita can induce neurotoxicity owing to their content of isoxazoles (Graeme 2014; Chen et al. 2016; White et al. 2019).

*Amanita muscaria*, distributed in the Northern Hemisphere, is the most representative neurotoxic species of sect. Amanita. For a long time, it was believed that the psychoactive effects of *A. muscaria* were due to muscarine, a toxin first isolated from this species (Schmiedeberg and Koppe 1869; Kögl et al. 1957). Muscarine is an alkaloid that binds to acetylcholine receptors and acts as a parasympathomimetic agent (Peredy and Bradford 2014). However, muscarine is present in *A. muscaria* at approximately 0.0003% by weight (Puschner 2018), a concentration that is insufficient to cause intoxication. In comparison, the concentration of muscarine is particularly high in species of *Clitocybaceae* and *Inocybaceae*, reaching up to 1.6% by weight (Genest et al. 1968; Deng et al. 2021; Li et al. 2021b; He et al. 2023). It is now recognized that, in *A. muscaria* mushrooms, ibotenic acid and its decarboxylation product, muscimol, are the primary psychoactive compounds (Widelski and Kukula-Koch 2017).

Ibotenic acid and muscimol are two isoxazoles initially isolated from *A. muscaria* (Takemoto et al. 1964; Mueller and Eugster 1965). They are structurally analogous to glutamic acid and gamma-aminobutyric acid (GABA), which can cause central nervous system symptoms by stimulating N-methyl-D-aspartate (NMDA) and GABA receptors (Wiegand 2024). Besides *A. muscaria*, these two toxins have been detected in 15 other species among the 112 globally accepted species within the sect. Amanita (Liu et al. 2022; Su et al. 2022, 2025; Zhou et al. 2023; https://www.amanitaceae.org, accessed on 20 May 2025). These species include *A. cothurnata* (Chilton and Ott 1976), *A. gemmata* (Benedict et al. 1966), *A. pantherina* (Tsujikawa et al. 2007), and *A. regalis* (https://www.mushroomthejournal.com, accessed on 20 May 2025) in Europe, and *A. ibotengutake* in Japan, as well as 10 of the 30 known sect. Amanita species in China: *A. altipes*, *A. concentrica*, *A. flavopantherina*, *A. griseopantherina*, *A. parvisychnopyramis*, *A. pseudopantherina*, *A. pseudosychnopyramis*, *A. rubrovolvata*, *A. subglobosa*, and *A. sychnopyramis* (Chen et al. 2022; Su et al. 2023, 2025).

Some other species of sect. Amanita, such as *A. collariata*, *A. melleiceps*, *A. melleialba*, *A. orientigemmata*, and *A. rufoferruginea* from East Asia (Li et al. 2022a, 2023, 2024), and *A. aprica* from North America (Tulloss and Lindgren 2005), have been reported to cause human intoxication with symptoms similar to those of *A. muscaria*. These species are also thought to contain ibotenic acid and muscimol. The presence of these toxins is generally considered to be confined to A. sect. Amanita. Species from this section have been responsible for a significant number of neurotoxic poisonings, including fatalities in Asia (Bau et al. 2025), Europe (Rampolli et al. 2021), and North America (Meisel et al. 2022) in recent years.

The biosynthesis of ibotenic acid and muscimol remained unclear until Obermaier and Müller (2020) elucidated two alternative biosynthetic pathways in *A. muscaria*. These pathways enable the conversion of the amino acids glutamine or glutamate into ibotenic acid and muscimol, involving seven core enzymes: IboA (adenylating), IboC (cytochrome P450), IboD (decarboxylase), IboF (flavin-dependent monooxygenase), IboG1 and *Ibo*G2 (two pyridoxal phosphate-dependent enzymes), and IboH (hydroxylase), whose genes occur in a biosynthetic gene cluster (BGC). BGCs are clusters of contiguous genes present in the genomes of bacteria, fungi, plants, and animals that are responsible for synthesizing secondary metabolites (Xia et al. 2022; Zhu et al. 2025). In fungi, the production of secondary metabolites via BGCs is commonly observed in the biosynthesis of many important compounds, such as the immunosuppressant cyclosporins, the cholesterol-reducing lovastatin, the antibiotic penicillins, the hallucinogenic prodrug psilocybin, and the mycotoxins trichothecenes and aflatoxins (Rokas et al. 2020).

In recent years, the rapid advancement of next-generation sequencing (NGS) technology has accelerated the generation of genomic data and significantly decreased associated costs. Based on genomic data, phylogenomic analysis has been increasingly used to resolve high-rank classifications of fungi, such as *Basidiomycota* (He et al. 2024), *Agaricales* (Wang et al. 2023), *Agaricineae*, *Pluteineae*, *Tricholomatineae* (Qu et al. 2025), *Clitocybaceae* (He et al. 2023), and *Cortinariaceae* (Liimatainen et al. 2022). Compared with mono- or multigenic phylogenetic datasets, genome-scale datasets provide a substantial increase in the statistical confidence of inferred relationships, yielding highly supported species trees (Shen et al. 2020; He et al. 2023). Genome sequencing has also enabled the discovery and identification of fungal BGCs (Tran et al. 2019), revealing the biosynthetic capacity and evolution of fungal secondary metabolites, as exemplified by the case study of the toxin psilocybin (Fricke et al. 2017; Awan et al. 2018; Bradshaw et al. 2024).

In our previous study (Su et al. 2023), we attempted to clarify the relationship between phylogeny and ibotenic acid and muscimol production within A. sect. Amanita. However, owing to unstable phylogenetic topology resulting from a limited number of loci and inconclusive toxin detection results caused by their instability, we failed to resolve the taxonomic distribution of ibotenic acid and muscimol within this section. In the present study, we address this issue by integrating toxin gene distribution into a phylogenomic analysis. Moreover, apart from *A. muscaria* and *A. pseudosychnopyramis* (Eugster and Schleusener 1969; Chen et al. 2022), the occurrence and concentration of muscarine in other species of this section remain largely unknown. Using 99 specimens representing 29 species from A. sect. Amanita, the present study aims (1) to reconstruct the phylogenetic framework of sect. Amanita using multilocus and genomic data, (2) to mine and identify ibo genes and detect muscarine in sect. Amanita species, (3) to investigate the distribution and estimate divergence times of the *ibo*BGC and muscarine within the sect. Amanita, and (4) to gain insight into the evolution of the *ibo*BGC by comparing gene and species trees.

## ﻿Methods

### ﻿Specimens

**Figure 1.**
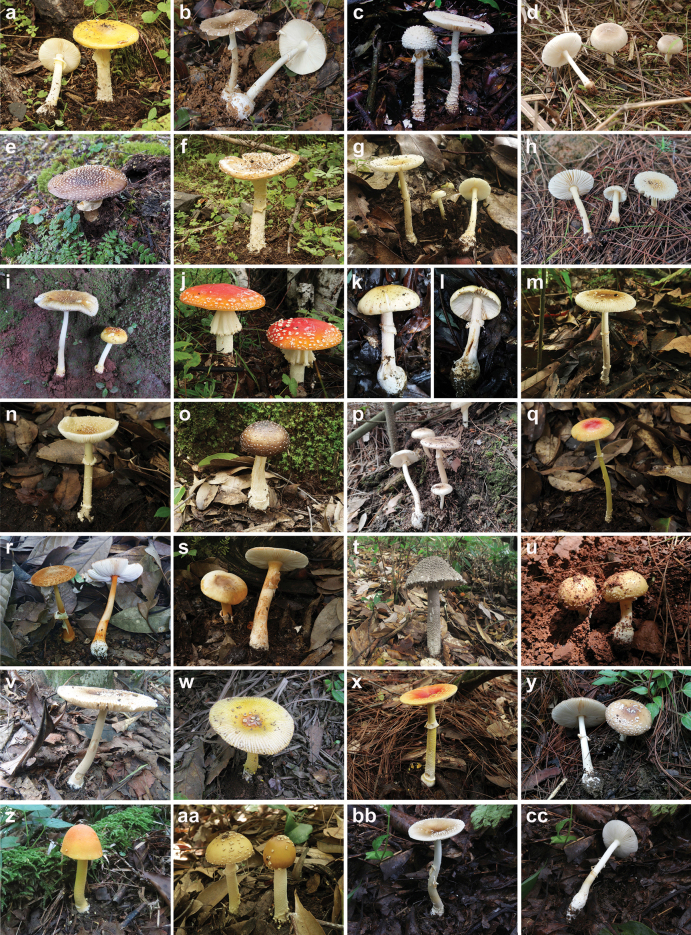
Basidiomata of representative species of Amanita sect. Amanita in China. **a***A. altipes* (MHHNU 33082); **b***A. collariata* (MHHNU 31095); **c***A. concentrica* (KUN-HKAS 84675, photographed by Li-Hong Han); **d***A. farinosa* (MHHNU 32768); **e***A. flavopantherina* (MHHNU 32267); **f***A. griseopantherina* (MHHNU 33084); **g***A. melleialba* (MHHNU 10606); **h***A. melleiceps* (MHHNU 32769); **i***A. mira* (KUN-HKAS 91953, photographed by Qing Cai); **j***A. muscaria* (MHHNU 32652); **k, l***A. orientigemmata* (MHHNU 32700); **m***A. parvipantherina* (MHHNU 9634); **n***A. parvisychnopyramis* (MHHNU 32953); **o***A. pseudopantherina* (MHHNU 32927); **p***A. pseudosychnopyramis* (MHHNU 32762); **q***A. rubrovolvata* (MHHNU 32949); **r***A. rufoferruginea* (MHHNU 30943); **s***A. siamensis* (MHHNU 32951); **t***A. sinensis* (MHHNU 8585); **u***Amanita* sp. 1 (MHHNU 31289); **v***Amanita* sp. 2 (MHHNU 30273); **w***Amanita* sp. 3 (MHHNU 30293); **x***A. subfrostiana* (MHHNU 32919); **y***A. subglobosa* (MHHNU 31308); **z***A. subparcivolvata* (MHHNU 33170); **aa***A. subparvipantherina* (MHHNU 32922); **bb, cc***A. sychnopyramis* (MHHNU 33272).

A total of 101 *Amanita* specimens were collected from Central (35), Eastern (4), Northeastern (6), Southern (1), and Southwestern (55) China between 2006 and 2022. Fresh basidiomata (Fig. 1) were subjected to heat-induced drying at 45°C. These specimens are currently preserved in the Mycological Herbarium of Hunan Normal University (MHHNU), Changsha, and the Herbarium of Kunming Institute of Botany, Chinese Academy of Sciences (KUN-HKAS), Kunming, China. Details regarding the specimens, including species names, herbarium numbers, collection dates and localities, and accession numbers, are provided in Tables 1–3.

**Table 1.** Determination of muscarine in Amanita species of sect. Amanita.

Species	Voucher	Accession number	Locality	Collection date	Muscarine (mg/kg)
*Amanita altipes*	MHHNU 32286	ON131731	China: Yunnan	Aug. 2020	–
	MHHNU 33050	C_AA120855	China: Sichuan	Aug. 2021	–
	MHHNU 33059	C_AA120866	China: Sichuan	Aug. 2021	–
	MHHNU 33071	C_AA120877	China: Sichuan	Aug. 2021	–
	MHHNU 33082	C_AA120881	China: Sichuan	Sep. 2021	–
*A. collariata*	MHHNU 31095	OM955206	China: Hunan	May 2018	–
*A. concentrica*	HKAS 84675	MH508326	China: Yunnan	Jul. 2014	6428.57
	HKAS 87061	MH508327	China: Yunnan	Jul. 2014	5009.35
*A. farinosa*	HKAS 56816	JN943180	China: Yunnan	Aug. 2009	–
	MHHNU 32693	ON131732	China: Hunan	Jul. 2020	–
	MHHNU 32768	ON131733	China: Hunan	May 2021	–
	MHHNU 32771	ON131734	China: Hunan	May 2021	–
*A. flavomelleiceps*	MHHNU 33119	PQ326880	China: Sichuan	Aug. 2021	–
*A. flavopantherina*	HKAS 51045	MH508353	China: Sichuan	Aug. 2006	1300.00
	HKAS 58795	MH508354	China: Yunnan	Aug. 2009	1544.23
	MHHNU 32267	ON131735	China: Yunnan	Aug. 2020	4000.00
*A. griseopantherina*	HKAS 58801	MH508381	China: Yunnan	Aug. 2009	–
	HKAS 82340	MH508383	China: Sichuan	Aug. 2013	–
	HKAS 83578	MH508386	China: Tibet	Jun. 2014	–
	MHHNU 33078	C_AA120882	China: Sichuan	Aug. 2021	–
	MHHNU 33084	C_AA120883	China: Sichuan	Aug. 2021	–
*A. ibotengutake*	HKAS 56045	MH508857	China: Jilin	Aug. 2008	–
	HKAS 83269	MH508858	China: Jilin	Aug. 2010	–
*A. melleialba*	MHHNU 10606	C_AA120884	China: Hunan	Jul. 2021	–
	MHHNU 31481	ON131736	China: Hunan	Jul. 2019	–
*A. melleiceps*	MHHNU 31865	ON131765	China: Hunan	May 2020	–
	MHHNU 32763	ON131767	China: Hunan	May 2021	–
	MHHNU 32769	ON131770	China: Hunan	May 2021	–
	MHHNU 32782	ON131768	China: Hunan	Jun. 2021	–
	MHHNU 32785	ON131769	China: Hunan	Jun. 2021	–
*A. mira*	HKAS 91953	MH508437	China: Yunnan	Jul. 2015	–
*A. muscaria*	MHHNU 7782	ON131737	China: Inner mongolia	Aug. 2013	473.68
	MHHNU 31764	ON131738	China: Inner mongolia	Sep. 2019	75.93
	MHHNU 32652	ON131739	China: Inner mongolia	Aug. 2020	84.41
	MHHNU 33653	C_AA120885	China: Inner mongolia	Aug. 2020	127.02
*A. orientigemmata*	HKAS 80978	MH508469	China: Yunnan	Aug. 2013	–
	MHHNU 32700	ON131764	China: Hunan	Jul. 2020	–
	MHHNU 33136	C_AA120886	China: Hunan	Aug. 2021	1.09
*A. parvipantherina*	MHHNU 9634	C_AA120856	China: Guizhou	Jun. 2018	–
	MHHNU 9637	C_AA120857	China: Guizhou	Jun. 2021	–
	MHHNU 32913	C_AA120858	China: Yunnan	Jul. 2021	–
	MHHNU 32942	C_AA120859	China: Yunnan	Jul. 2021	–
	MHHNU 32960	C_AA120860	China: Yunnan	Jul. 2021	–
*A. parvisychnopyramis*	MHHNU 11290	PQ326875	China: Yunnan	Aug. 2022	1777.36
	MHHNU 32953	PQ326876	China: Yunnan	Jul. 2021	897.20
*A. pseudopantherina*	HKAS 57611	MH508511	China: Yunnan	Aug. 2009	–
	HKAS 83636	MH508515	China: Yunnan	Aug. 2014	–
	MHHNU 11333	C_AA120861	China: Yunnan	Aug. 2022	–
	MHHNU 31028	ON131744	China: Yunnan	Aug. 2017	–
	MHHNU 32927	C_AA120862	China: Yunnan	Jul. 2021	–
*A. pseudosychnopyramis*	HKAS 78417	MH508528	China: Yunnan	Mar. 2012	1556.19
	HKAS 82293	MH508529	China: Yunnan	Mar. 2014	1027.27
	MHHNU 32762	MZ313998	China: Zhejiang	Apr. 2021	1438.66
*A. rubrovolvata*	MHHNU 9582	C_AA120863	China: Guizhou	Jun. 2018	–
	MHHNU 31903	ON131748	China: Hunan	Jun. 2020	–
	MHHNU 32265	ON131749	China: Yunnan	Aug. 2020	–
	MHHNU 32524	ON131751	China: Hunan	Sep. 2020	–
	MHHNU 32949	C_AA120864	China: Yunnan	Jul. 2021	–
*A. rufoferruginea*	MHHNU 10663	C_AA120865	China: Hainan	Jul. 2021	–
	MHHNU 30943	ON131771	China: Hunan	Jun. 2015	–
	MHHNU 31046	ON131772	China: Hunan	Jun. 2017	–
	MHHNU 31100	ON131773	China: Hunan	Jun. 2018	–
	MHHNU 31887	ON131774	China: Hunan	Jun. 2020	–
*A. siamensis*	HKAS 67855	MH508592	China: Yunnan	Aug. 2010	–
	HKAS 83681	MH508593	China: Yunnan	Aug. 2014	–
	MHHNU 30976	ON131775	China: Hunan	Jul. 2016	–
	MHHNU 31578	ON131776	China: Yunnan	Aug. 2019	–
	MHHNU 32951	C_AA120867	China: Yunnan	Jul. 2021	–
*A. sinensis*	HKAS 80012	MH508597	China: Yunnan	Aug. 2013	–
	HKAS 100492	MH508594	China: Fujian	Aug. 2013	–
	MHHNU 8585	MK239263	China: Hunan	Aug. 2015	–
*Amanita* sp. 1	MHHNU 31289	C_AA120868	China: Yunnan	Aug. 2018	–
*Amanita* sp. 2	MHHNU 30273	C_AA120869	China: Hunan	Jun. 2007	–
*Amanita* sp. 3	MHHNU 30293	C_AA120870	China: Hunan	Jun. 2009	–
*A. subfrostiana*	HKAS 58847	JN943172	China: Yunnan	Aug. 2009	780.95
	HKAS 76308	MH508616	China: Sichuan	Jul. 2012	1366.67
	MHHNU 11320	C_AA120871	China: Yunnan	Aug. 2022	713.33
	MHHNU 32919	C_AA120872	China: Yunnan	Jul. 2021	807.34
	MHHNU 32955	C_AA120873	China: Yunnan	Jul. 2021	700.95
*A. subglobosa*	MHHNU 11257	C_AA120874	China: Yunnan	Aug. 2022	–
	MHHNU 31083	MK388157	China: Hunan	May 2018	–
	MHHNU 31084	MK239245	China: Hunan	May 2018	–
	MHHNU 31308	ON131752	China: Yunnan	Aug. 2018	–
	MHHNU 32538	ON131753	China: Hunan	Sep. 2020	–
*A. subparcivolvata*	MHHNU 31557	OM955212	China: Hubei	Aug. 2019	–
	MHHNU 32368	OM955213	China: Hunan	Jul. 2020	–
	MHHNU 32849	OM955210	China: Hunan	Jul. 2021	–
	MHHNU 32907	OM955216	China: Hunan	Jul. 2021	–
	MHHNU 33170	OM955215	China: Hunan	Jul. 2021	–
*A. subparvipantherina*	MHHNU 11319	C_AA120875	China: Yunnan	Aug. 2022	–
	MHHNU 32163	ON131754	China: Yunnan	Aug. 2020	–
	MHHNU 32226	ON131756	China: Yunnan	Aug. 2020	–
	MHHNU 32354	ON131758	China: Yunnan	Aug. 2020	–
	MHHNU 32922	C_AA120876	China: Yunnan	Jul. 2021	–
*A. sychnopyramis*	MHHNU 32153	ON131761	China: Anhui	Aug. 2020	–
	MHHNU 32799	ON131762	China: Zhejiang	Jul. 2021	–
	MHHNU 33272	C_AA120878	China: Hunan	Jul. 2022	–
	MHHNU 33330	C_AA120879	China: Hunan	Jul. 2022	–
	MHHNU 33350	C_AA120880	China: Hunan	Jul. 2022	–
*A. avellaneifolia* (sect. Roanokenses)	MHHNU 33518	PX598993	China: Hunan	Jul. 2022	–
*A. flavipes* (sect. Validae)	MHHNU 32280	PX587958	China: Yunnan	Aug. 2020	–

The content of muscarine is presented on a dry-weight basis, and “–” indicates that the toxin was not detected in the sample, meaning that the concentration was below the limit of detection. Sequences with C_AA000000 are publicly accessible in GenBase, whereas the remaining sequences are accessible in GenBank.

**Table 2.** Sequences used for two-locus phylogenetic analysis of *Amanita*.

Section	Species	Voucher	Locality	ITS	LSU	Reference
* Amanita *	*Amanita albocreata*	RET 547-7	Canada	KU248128	–	GenBank
	*A. alpinicola*	MONT CLC2376 (holotype)	USA	NR158316	–	Cripps et al. 2017
	*A. alpinicola*	RET 888-8	USA	OL584339	OL584339	GenBank
	*A. altipes*	HKAS 36609 (holotype)	China	AY436445	–	Zhang et al. 2004
	*A. altipes*	MHHNU 32286	China	ON131731	ON139026	Su et al. 2023
	*A. altipes*	MHHNU 33050	China	**C_AA120855**	**C_AA120982**	Present study
	*A. altipes*	MHHNU 33059	China	**C_AA120866**	**C_AA120993**	Present study
	*A. altipes*	MHHNU 33071	China	**C_AA120877**	**C_AA121003**	Present study
	*A. altipes*	MHHNU 33082	China	**C_AA120881**	**C_AA121007**	Present study
	*A.* ‘*amerlpanthera*’	RET 387-10	USA	MT445432	HQ539707	GenBank
	*A. aprica*	RET 128-5 (isotype)	USA	NR154683	NG057029	Tulloss and Lindgren 2005
	*A.* ‘*austrowellsii*’	RET 302-1	USA	MN963578	MN963578	GenBank
	*A. bingensis*	KM 104 (epitype)	Cameroon	MT446264	MT446281	Mighell et al. 2021
	*A. breckonii*	NY 00066695 (isotype)	USA	KJ535439	KJ535440	GenBank
	A. cf. albopulverulenta	KM 48	Cameroon	–	MT446282	Mighell et al. 2021
	*A. chrysoblema*	RET 320-1	USA	EU071911	EU071984	Geml et al. 2008
	*A. collariata*	MHHNU 31095 (holotype)	China	OM955206	OM955204	Su et al. 2022
	*A. concentrica*	CBM FB-24901 (holotype)	Japan	NR119387	–	Oda et al. 2002
	*A. concentrica*	HKAS 84675	China	MH508326	MH486474	Cui et al. 2018
	*A. concentrica*	HKAS 87061	China	MH508327	KR824785	Cui et al. 2018
	*A. crenulata*	RET 675-9	USA	MK204468	MK204474	GenBank
	*A. cruzii*	BARONI 8998 (paratype)	Dominican Republic	KC855222	KC855222	Miller and Lodge 2001
	*A. diemii*	MES 1335	Argentina	–	KY053387	Truong et al. 2017
	*A. digitosa*	BBH 32154 (holotype)	Thailand	KT213722	–	Li et al. 2016
	*A. elata*	HKAS 83449	China	MH508334	MH486486	Cui et al. 2018
	*A. farinosa*	iNAT 136301871 /RET534-10	USA	OP749631	KU186823	GenBank
	*A. farinosa*	HKAS 56816	China	JN943180	JN941154	Schoch et al. 2012
	*A. farinosa*	MHHNU 32693	China	ON131732	ON139027	Su et al. 2023
	*A. farinosa*	MHHNU 32768	China	ON131733	ON139028	Su et al. 2023
	*A. farinosa*	MHHNU 32771	China	ON131734	ON139029	Su et al. 2023
	*A. fibrillopes*	PERTH08793573	Australia	MN918103	MN918099	Davison et al. 2020
* Amanita *	*A. flavoalba*	CAL 1405 (holotype)	India	–	KY861748	Phookamsak et al. 2019
	*A. flavomelleiceps*	MHHNU 33119 (holotype)	China	PQ326880	PQ330908	Su et al. 2025
	*A. flavopantherina*	HKAS 51045	China	MH508353	MH486517	Cui et al. 2018
	*A. flavopantherina*	HKAS 58795	China	MH508354	MH486518	Cui et al. 2018
	*A. flavopantherina*	HKAS 82613 (holotype)	China	MH508355	MH486519	Cui et al. 2018
	*A. flavopantherina*	MHHNU 32267	China	ON131735	ON139030	Su et al. 2023
	*A. frostiana*	RET 543-6 /RET 7-25-92 E	Canada	KP313580	AF024453	Weiss et al. 1998
	*A. gemmata*	JM96/62	England	–	AF097371	Drehmel et al. 1999
	*A. gioiosa*	ML61952AG	Cyprus	MH603599	–	Loizides et al. 2018
	*A. gleocystidiosa*	BBH 31903 (holotype)	Thailand	KT213719	–	Li et al. 2016
	*A. griseopantherina*	HKAS 58801	China	MH508381	MH486569	Cui et al. 2018
	*A. griseopantherina*	HKAS 82340	China	MH508383	MH486571	Cui et al. 2018
	*A. griseopantherina*	HKAS 83560 (holotype)	China	MH508385	MH486573	Cui et al. 2018
	*A. griseopantherina*	HKAS 83578	China	MH508386	MH486574	Cui et al. 2018
	*A. griseopantherina*	MHHNU 33078	China	**C_AA120882**	**C_AA121008**	Present study
	*A. griseopantherina*	MHHNU 33084	China	**C_AA120883**	**C_AA121009**	Present study
	*A.* ‘*hallingiana*’	RET 292-7	Mexico	KY924833	–	GenBank
	*A. ibotengutake*	CBM FB-30969 (holotype)/30974	Japan	NR119388	AB088767	Oda et al. 2002; Schoch et al. 2012
	*A. ibotengutake*	HKAS 56045	China	–	MH486589	Cui et al. 2018
	*A. ibotengutake*	HKAS 83269	China	–	MH486590	Cui et al. 2018
	*A. kalasinensis*	CMUB-39966 (holotype)	Thailand	OM040562	OM040553	Liu et al. 2022
	*A. melleialba*	HKAS 83446 (holotype)	China	MH508430	KR824767	Cui et al. 2018
	*A. melleialba*	MHHNU 10606	China	**C_AA120884**	**C_AA121010**	Present study
	*A. melleialba*	MHHNU 31481	China	ON131736	ON139031	Su et al. 2023
	*A. melleiceps*	CBM FB-30953	Japan	AB015688	–	Oda et al. 2002
	*A. melleiceps*	MHHNU 31865	China	ON131765	ON139065	Su et al. 2023
	*A. melleiceps*	MHHNU 32763	China	ON131767	ON139067	Su et al. 2023
	*A. melleiceps*	MHHNU 32769	China	ON131770	ON139070	Su et al. 2023
	*A. melleiceps*	MHHNU 32782	China	ON131768	ON139068	Su et al. 2023
	*A. melleiceps*	MHHNU 32785	China	ON131769	ON139069	Su et al. 2023
	*A. minima*	KM 38 (holotype)	Cameroon	MT446261	MT446280	Mighell et al. 2021
	*A. mira*	HKAS 91953	China	MH508437	MH486646	Cui et al. 2018
	*A. multisquamosa*	RET 374-1/BW RP14	USA	MK840916	HQ539710	Hughes et al. 2020
	*A. muscaria* *	MB-001171	Germany	MH508442	MH486652	Cui et al. 2018
	*A. muscaria* *	MHHNU 7782	China	ON131737	ON139032	Su et al. 2023
	*A. muscaria* *	MHHNU 31764	China	ON131738	ON139033	Su et al. 2023
	*A. muscaria* *	MHHNU 32652	China	ON131739	ON139034	Su et al. 2023
	*A. muscaria* *	MHHNU 33653	China	**C_AA120885**	**C_AA121011**	Present study
	*A. nehuta*	JAC10720	New Zealand	MT863749	MT862256	GenBank
	*A. orientigemmata*	HKAS 80978	China	MH508469	MH486708	Cui et al. 2018
	*A. orientigemmata*	MHHNU 32700	China	ON131764	ON139063	Su et al. 2023
	*A. orientigemmata*	MHHNU 33136	China	**C_AA120886**	**C_AA121012**	Present study
	*A. pakistanica*	RET 317-6	Pakistan	KX365198	KX365199	GenBank
	*A. pantherina*	MB-102863	Germany	MH508488	MH486743	Cui et al. 2018
	*A. pantherinoides*	iNAT 25463910	USA	OM212868	–	GenBank
* Amanita *	*A. parcivolvata*	RET 614-4	USA	MN963585	MN963584	GenBank
	*A. parvipantherina*	HKAS 83663	China	MH508499	MH486752	Cui et al. 2018
	*A. parvipantherina*	MHHNU 9634	China	**C_AA120856**	**C_AA120983**	Present study
	*A. parvipantherina*	MHHNU 9637	China	**C_AA120857**	**C_AA120984**	Present study
	*A. parvipantherina*	MHHNU 32913	China	**C_AA120858**	**C_AA120985**	Present study
	*A. parvipantherina*	MHHNU 32942	China	**C_AA120859**	**C_AA120986**	Present study
	*A. parvipantherina*	MHHNU 32960	China	**C_AA120860**	**C_AA120987**	Present study
	*A. parvisychnopyramis*	MHHNU 11290	China	PQ326875	PQ326881	Su et al. 2025
	*A. parvisychnopyramis*	MHHNU 32953 (holotype)	China	PQ326876	PQ330907	Su et al. 2025
	*A. persicina*	RET 151-4	USA	EU071892	EU071969	Geml et al. 2008
	*A.* ‘*praecox*’	BW-PH082906-9	USA	–	HQ539725	GenBank
	*A. pseudopantherina*	HKAS 57611	China	MH508511	MH486774	Cui et al. 2018
	*A. pseudopantherina*	HKAS 80007 (holotype)	China	MH508514	MH486777	Cui et al. 2018
	*A. pseudopantherina*	HKAS 83636	China	MH508515	MH486778	Cui et al. 2018
	*A. pseudopantherina*	MHHNU 11333	China	**C_AA120861**	**C_AA120988**	Present study
	*A. pseudopantherina*	MHHNU 31028	China	ON131744	ON139039	Su et al. 2023
	*A. pseudopantherina*	MHHNU 32927	China	**C_AA120862**	**C_AA120989**	Present study
	*A. pseudosychnopyramis*	HKAS 78417	China	MH508528	KR824777	Cui et al. 2018
	*A. pseudosychnopyramis*	HKAS 82293	China	MH508529	MH486790	Cui et al. 2018
	*A. pseudosychnopyramis*	HKAS 87999 (holotype)	China	MH508530	KR824778	Cui et al. 2018
	*A. pseudosychnopyramis*	MHHNU 32762	China	MZ 313998	OM753110	Su et al. 2023
	*A. pudica*	RET 344-40	Zambia	–	HQ539730	GenBank
	*A. pubescens*	RET 802-10	USA	MH930991	MH931002	GenBank
	*A. pyriformis*	BBH 38643 (holotype)	Thailand	KT213723	–	Li et al. 2016
	*A. ravicrocina*	CMUB 39967 (holotype)	Thailand	OM040568	OM040559	Liu et al. 2022
	*A. regalis*	HKAS 56699	Czech Republic	MH508537	MH486797	Cui et al. 2018
	*A. rubrovolvata*	CBM FB-30980	Japan	AB096053	–	Oda et al. 2002
	*A. rubrovolvata*	MHHNU 9582	China	**C_AA120863**	**C_AA120990**	Present study
	*A. rubrovolvata*	MHHNU 31903	China	ON131748	ON139043	Su et al. 2023
	*A. rubrovolvata*	MHHNU 32265	China	ON131749	ON139044	Su et al. 2023
	*A. rubrovolvata*	MHHNU 32524	China	ON131751	ON139046	Su et al. 2023
	*A. rubrovolvata*	MHHNU 32949	China	**C_AA120864**	**C_AA120991**	Present study
	*A. rufoferruginea*	HKAS 84974	China	MH508580	MH486843	Cui et al. 2018
	*A. rufoferruginea*	MHHNU 10663	China	**C_AA120865**	**C_AA120992**	Present study
	*A. rufoferruginea*	MHHNU 30943	China	ON131771	ON139071	Su et al. 2023
	*A. rufoferruginea*	MHHNU 31046	China	ON131772	ON139072	Su et al. 2023
	*A. rufoferruginea*	MHHNU 31100	China	ON131773	ON139073	Su et al. 2023
	*A. rufoferruginea*	MHHNU 31887	China	ON131774	ON139074	Su et al. 2023
	*A. robusta*	YA KM 41 (epitype)	Cameroon	NR173187	MT446278	Mighell et al. 2021
	*A. roseotincta*	RET 313-5	USA	MH508551	–	Cui et al. 2018
	*A. siamensis*	HKAS 67855	China	MH508592	MH486864	Cui et al. 2018
	*A. siamensis*	HKAS 83681	China	MH508593	MH486866	Cui et al. 2018
	*A. siamensis*	MHHNU 30976	China	ON131775	ON139076	Su et al. 2023
	*A. siamensis*	MHHNU 31578	China	ON131776	–	Su et al. 2023
	*A. siamensis*	MHHNU 32951	China	**C_AA120867**	**C_AA120994**	Present study
	*A. sinensis*	HKAS 80012	China	MH508597	MH486871	Cui et al. 2018
* Amanita *	*A. sinensis*	HKAS 100492	China	MH508594	MH486867	Cui et al. 2018
	*A. sinensis*	MHHNU 8585	China	MK239263	ON139047	Su et al. 2023
	*Amanita* sp. 1	MHHNU 31289	China	**C_AA120868**	**C_AA120995**	Present study
	*Amanita* sp. 2	MHHNU 30273	China	**C_AA120869**	**C_AA120996**	Present study
	*Amanita* sp. 3	MHHNU 30293	China	**C_AA120870**	–	Present study
	*A. stranella*	TENN 60935	USA	FJ596814	–	Hughes et al. 2009
	*A. subfrostiana*	HKAS 32513 (holotype)	China	–	AF024477	Weiss et al. 1998
	*A. subfrostiana*	HKAS 58847	China	JN943172	JN941161	Schoch et al. 2012
	*A. subfrostiana*	HKAS 76308	China	MH508616	MH486898	Cui et al. 2018
	*A. subfrostiana*	MHHNU 11320	China	**C_AA120871**	**C_AA120997**	Present study
	*A. subfrostiana*	MHHNU 32919	China	**C_AA120872**	**C_AA120998**	Present study
	*A. subfrostiana*	MHHNU 32955	China	**C_AA120873**	**C_AA120999**	Present study
	*A. subglobosa*	HKAS 12009 (holotype)	China	–	AF024478	Weiss et al. 1998
	*A. subglobosa*	MHHNU 11257	China	**C_AA120874**	**C_AA121000**	Present study
	*A. subglobosa*	MHHNU 31083	China	MK388157	ON139048	Su et al. 2023
	*A. subglobosa*	MHHNU 31084	China	MK239245	ON139049	Su et al. 2023
	*A. subglobosa*	MHHNU 31308	China	ON131752	ON139050	Su et al. 2023
	*A. subglobosa*	MHHNU 32538	China	ON131753	ON139051	Su et al. 2023
	*A. submelleialba*	CMUB S1 (holotype)	Thailand	NR184934	NG088232	Liu et al. 2021
	*A. subparcivolvata*	MHHNU 31557	China	OM955212	OM955208	Su et al. 2022
	*A. subparcivolvata*	MHHNU 32368	China	OM955213	OM955211	Su et al. 2022
	*A. subparcivolvata*	MHHNU 32849	China	OM955210	OM955209	Su et al. 2022
	*A. subparcivolvata*	MHHNU 32907 (holotype)	China	OM955216	OM955205	Su et al. 2022
	*A. subparcivolvata*	MHHNU 33170	China	OM955215	OM955214	Su et al. 2022
	*A. subparvipantherina*	HKAS 56986 (holotype)	China	–	KR824776	GenBank
	*A. subparvipantherina*	MHHNU 11319	China	**C_AA120875**	**C_AA121001**	Present study
	*A. subparvipantherina*	MHHNU 32163	China	ON131754	ON139051	Su et al. 2023
	*A. subparvipantherina*	MHHNU 32226	China	ON131756	ON139054	Su et al. 2023
	*A. subparvipantherina*	MHHNU 32354	China	ON131758	ON139056	Su et al. 2023
	*A. subparvipantherina*	MHHNU 32922	China	**C_AA120876**	**C_AA121002**	Present study
	*A.* ‘*subvelatipes*’	RET 483-10	USA	MK204467	–	GenBank
	*A. sychnopyramis*	HKAS 75485	China	MH508634	MH486926	Cui et al. 2018
	*A. sychnopyramis*	MHHNU 32153	China	ON131761	ON139060	Su et al. 2023
	*A. sychnopyramis*	MHHNU 32799	China	ON131762	ON139061	Su et al. 2023
	*A. sychnopyramis*	MHHNU 33272	China	**C_AA120878**	**C_AA121004**	Present study
	*A. sychnopyramis*	MHHNU 33330	China	**C_AA120879**	**C_AA121005**	Present study
	*A. sychnopyramis*	MHHNU 33350	China	**C_AA120880**	**C_AA121006**	Present study
	*A. taiepa*	JAC12826	New Zealand	MT863755	MT862263	GenBank
	*A.* ‘*tlaxcalipanthera*’	Mushroom Observer 428571	USA	MW633035	–	GenBank
	*A. velatipes*	RET 489-2	USA	MH508643	MH486938	Cui et al. 2018
	*A. viscidolutea*	ANMF 767	Brazil	MW000473	–	GenBank
	*A. wellsii*	RET 259-1	USA	KU248116	OK285331	GenBank
	*A. xylinivolva*	ANDES F312 NVE56	Colombia	FJ890024	FJ890036	Vargas et al. 2011
* Amarrendiae *	*A. oleosa* *	H7627	Australia	GQ925400	GQ925377	Justo et al. 2010
	*A. umbrinella*	PSC 1813	Australia	AY194981	HQ539753	GenBank
* Amidella *	*A. pinophila*	HKAS 68307	China	MH508503	MH486758	Cui et al. 2018
	*A. volvata* *	S. Harsch 304	USA	–	AF024485	Weiss et al. 1998
* Arenariae *	*A. arenaria* *	VPI679	Australia	–	GQ925382	Justo et al. 2010
* Caesareae *	*A. caesarea* *	HKAS 96166	Italy	MH508283	MH486418	Cui et al. 2018
	*A. rubroflava*	HKAS 83089	China	MH508568	MH486827	Cui et al. 2018
* Phalloideae *	*A. exitialis*	HKAS 75775	China	JX998026	JX998053	Cai et al. 2012
	*A. phalloides* *	HKAS 75773	USA	JX998031	JX998060	Cai et al. 2012
* Roanokenses *	*A. caojizong*	HKAS 56933	China	KJ466378	KJ466438	Cai et al. 2014
	*A. neoovoidea*	HKAS 89025/100506	China	MH508445	MH486654	Cui et al. 2018
* Strobiliformes *	*A. cinereoradicata*	HKAS 101435	China	MH508307	MH486451	Cui et al. 2018
	*A. strobiliformis* *	MB-001177	Germany	MH508614	MH486895	Cui et al. 2018
* Vaginatae *	*A. griseofolia*	HKAS 96928	China	MH508379	MH486567	Cui et al. 2018
	*A. liquii*	HKAS 93915	China	MH508427	MH486628	Cui et al. 2018
* Validae *	*A. excelsa* *	HKAS 31510	Germany	AY436453	AY436491	Zhang et al. 2004
	*A. flavipes*	HKAS 87944	China	MH508344	MH486507	Cui et al. 2018
* Lepidella *	*A. flavofloccosa*	HKAS 90174/92006	China	MH508352	MH486516	Cui et al. 2018
	*A. vittadinii*	HKAS 101430	Italy	MH508651	MH486950	Cui et al. 2018

The newly generated sequences submitted to GenBase are highlighted in bold, and the type species of each section are marked with an asterisk.

**Table 3.** Sequences used for five-locus phylogenetic analysis of sect. Amanita.

Species	Voucher	ITS	LSU	*RPB2*	*TEF1*	*TUB*	Reference
*Amanita altipes*	MHHNU 32286	ON131731	ON139026	ON229622	ON229662	ON125308	Su et al. 2023
*A. altipes*	MHHNU 33050	**C_AA120855**	**C_AA120982**	**C_AA121013**	**C_AA121135**	**C_AA121164**	Present study
*A. altipes*	MHHNU 33059	**C_AA120866**	**C_AA120993**	**C_AA121023**	**C_AA121145**	**C_AA121174**	Present study
*A. altipes*	MHHNU 33071	**C_AA120877**	**C_AA121003**	**C_AA121032**	**C_AA121154**	**C_AA121185**	Present study
*A. altipes*	MHHNU 33082	**C_AA120881**	**C_AA121007**	**C_AA121036**	**C_AA121158**	**C_AA121189**	Present study
*A. collariata*	MHHNU 31095	OM955206	OM955204	OM949814	OM949813	OM949821	Su et al. 2022
*A. concentrica*	HKAS 84675	MH508326	MH486474	MH485953	MH508749	MH485476	Cui et al. 2018
*A. concentrica*	HKAS 87061	MH508327	KR824785	KR824794	KR824827	MH485477	Cui et al. 2018
*A. farinosa*	HKAS 56816	JN943180	JN941154	JQ031110	MH508772	MH485496	Schoch et al. 2012; Cui et al. 2018
*A. farinosa*	MHHNU 32693	ON131732	ON139027	ON229623	ON229663	ON125309	Su et al. 2023
*A. farinosa*	MHHNU 32768	ON131733	ON139028	ON229624	ON229664	ON125310	Su et al. 2023
*A. farinosa*	MHHNU 32771	ON131734	ON139029	ON229625	ON229665	ON125311	Su et al. 2023
*A. flavomelleiceps*	MHHNU 33119	PQ326880	PQ330908	PQ356792	PQ356798	PQ356795	Su et al. 2025
*A. flavopantherina*	HKAS 51045	MH508353	MH486517	MH485988	MH508793	MH485510	Cui et al. 2018
*A. flavopantherina*	HKAS 58795	MH508354	MH486518	–	MH508794	MH485511	Cui et al. 2018
*A. flavopantherina*	MHHNU 32267	ON131735	ON139030	ON229626	ON229666	ON125312	Su et al. 2023
*A. griseopantherina*	HKAS 58801	MH508381	MH486569	MH486033	–	MH485552	Cui et al. 2018
*A. griseopantherina*	HKAS 82340	MH508383	MH486571	MH486035	MH508840	MH485554	Cui et al. 2018
*A. griseopantherina*	HKAS 83578	MH508386	MH486574	MH486037	MH508843	MH485557	Cui et al. 2018
*A. griseopantherina*	MHHNU 33078	**C_AA120882**	**C_AA121008**	**C_AA121037**	**C_AA121159**	**C_AA121190**	Present study
*A. griseopantherina*	MHHNU 33084	**C_AA120883**	**C_AA121009**	**C_AA121038**	**C_AA121160**	**C_AA121191**	Present study
*A. ibotengutake*	HKAS 56045	–	MH486589	–	MH508857	–	Cui et al. 2018
*A. ibotengutake*	HKAS 83269	–	MH486590	MH486051	MH508858	MH485570	Cui et al. 2018
*A. melleialba*	MHHNU 10606	**C_AA120884**	**C_AA121010**	**C_AA121039**	**C_AA121161**	**C_AA121192**	Present study
*A. melleialba*	MHHNU 31481	ON131736	ON139031	ON229627	ON229667	ON125313	Su et al. 2023
*A. melleiceps*	MHHNU 31865	ON131765	ON139065	ON262923	ON262909	ON125347	Su et al. 2023
*A. melleiceps*	MHHNU 32763	ON131767	ON139067	ON262925	ON262911	ON125349	Su et al. 2023
*A. melleiceps*	MHHNU 32769	ON131770	ON139070	ON262928	ON262914	ON125352	Su et al. 2023
*A. melleiceps*	MHHNU 32782	ON131768	ON139068	ON262926	ON262912	ON125350	Su et al. 2023
*A. melleiceps*	MHHNU 32785	ON131769	ON139069	ON262927	ON262913	ON125351	Su et al. 2023
*A. mira*	HKAS 91953	MH508437	MH486646	MH486097	–	–	Cui et al. 2018
*A. muscaria*	MHHNU 7782	ON131737	ON139032	ON229628	ON229668	ON125314	Su et al. 2023
*A. muscaria*	MHHNU 31764	ON131738	ON139033	ON229629	ON229669	ON125315	Su et al. 2023
*A. muscaria*	MHHNU 32652	ON131739	ON139034	ON229630	ON229670	ON125316	Su et al. 2023
*A. muscaria*	MHHNU 33653	**C_AA120885**	**C_AA121011**	**C_AA121040**	**C_AA121162**	**C_AA121193**	Present study
*A. orientigemmata*	HKAS 80978	MH508469	MH486708	MH486140	MH508948	MH485649	Cui et al. 2018
*A. orientigemmata*	MHHNU 32700	ON131764	ON139063	ON229657	ON229661	ON125345	Su et al. 2023
*A. orientigemmata*	MHHNU 33136	**C_AA120886**	**C_AA121012**	**C_AA121041**	**C_AA121163**	**C_AA121194**	Present study
*A. parvipantherina*	MHHNU 9634	**C_AA120856**	**C_AA120983**	**C_AA121014**	**C_AA121136**	**C_AA121165**	Present study
*A. parvipantherina*	MHHNU 9637	**C_AA120857**	**C_AA120984**	**C_AA121015**	**C_AA121137**	**C_AA121166**	Present study
*A. parvipantherina*	MHHNU 32913	**C_AA120858**	**C_AA120985**	**C_AA121016**	**C_AA121138**	**C_AA121167**	Present study
*A. parvipantherina*	MHHNU 32942	**C_AA120859**	**C_AA120986**	**C_AA121017**	**C_AA121139**	**C_AA121168**	Present study
*A. parvipantherina*	MHHNU 32960	**C_AA120860**	**C_AA120987**	**C_AA121018**	**C_AA121140**	**C_AA121169**	Present study
*A. parvisychnopyramis*	MHHNU 11290	PQ326875	PQ326881	PQ356790	PQ356796	PQ356793	Su et al. 2025
*A. parvisychnopyramis*	MHHNU 32953	PQ326876	PQ330907	PQ356791	PQ356797	PQ356794	Su et al. 2025
*A. pseudopantherina*	HKAS 57611	MH508511	MH486774	MH486188	MH509001	MH485695	Cui et al. 2018
*A. pseudopantherina*	HKAS 83636	MH508515	MH486778	MH486192	MH509005	MH485699	Cui et al. 2018
*A. pseudopantherina*	MHHNU 11333	**C_AA120861**	**C_AA120988**	**C_AA121019**	**C_AA121141**	**C_AA121170**	Present study
*A. pseudopantherina*	MHHNU 31028	ON131744	ON139039	ON229635	ON22967	ON125321	Su et al. 2023
*A. pseudopantherina*	MHHNU 32927	**C_AA120862**	**C_AA120989**	**C_AA121020**	**C_AA121142**	**C_AA121171**	Present study
*A. pseudosychnopyramis*	HKAS 78417	MH508528	KR824777	–	–	–	Cui et al. 2018
*A. pseudosychnopyramis*	HKAS 82293	MH508529	MH486790	MH486204	MH509016	MH485712	Cui et al. 2018
*A. pseudosychnopyramis*	MHHNU 32762	MZ 313998	OM753110	OM777286	OM777284	OM777285	Su et al. 2023
*A. rubrovolvata*	MHHNU 9582	**C_AA120863**	**C_AA120990**	–	–	**C_AA121172**	Present study
*A. rubrovolvata*	MHHNU 31903	ON131748	ON139043	ON229639	ON229679	ON125325	Su et al. 2023
*A. rubrovolvata*	MHHNU 32265	ON131749	ON139044	ON229640	ON229680	ON125326	Su et al. 2023
*A. rubrovolvata*	MHHNU 32524	ON131751	ON139046	ON229642	ON229682	ON125328	Su et al. 2023
*A. rubrovolvata*	MHHNU 32949	**C_AA120864**	**C_AA120991**	**C_AA121021**	**C_AA121143**	–	Present study
*A. rufoferruginea*	MHHNU 10663	**C_AA120865**	**C_AA120992**	**C_AA121022**	**C_AA121144**	**C_AA121173**	Present study
*A. rufoferruginea*	MHHNU 30943	ON131771	ON139071	ON262929	ON262915	ON125353	Su et al. 2023
*A. rufoferruginea*	MHHNU 31046	ON131772	ON139072	ON262930	ON262916	ON125354	Su et al. 2023
*A. rufoferruginea*	MHHNU 31100	ON131773	ON139073	ON262931	ON262917	ON125355	Su et al. 2023
*A. rufoferruginea*	MHHNU 31887	ON131774	ON139074	ON262932	ON262918	ON125356	Su et al. 2023
*A. siamensis*	HKAS 67855	MH508592	MH486864	MH486271	MH509087	MH485773	Cui et al. 2018
*A. siamensis*	HKAS 83681	MH508593	MH486866	MH486273	MH509089	MH485774	Cui et al. 2018
*A. siamensis*	MHHNU 30976	ON131775	ON139076	ON262934	ON262920	ON125358	Su et al. 2023
*A. siamensis*	MHHNU 31578	ON131776	–	ON262935	ON262921	ON125359	Su et al. 2023
*A. siamensis*	MHHNU 32951	**C_AA120867**	**C_AA120994**	**C_AA121024**	**C_AA121146**	**C_AA121175**	Present study
*A. sinensis*	HKAS 80012	MH508597	MH486871	–	–	–	Cui et al. 2018
*A. sinensis*	HKAS 100492	MH508594	MH486867	MH486274	MH509090	MH485775	Cui et al. 2018
*A. sinensis*	MHHNU 8585	MK239263	ON139047	ON229643	ON229683	ON125329	Su et al. 2023
*Amanita* sp. 1	MHHNU 31289	**C_AA120868**	**C_AA120995**	**C_AA121025**	**C_AA121147**	**C_AA121176**	Present study
*Amanita* sp. 2	MHHNU 30273	**C_AA120869**	**C_AA120996**	–	–	**C_AA121177**	Present study
*Amanita* sp. 3	MHHNU 30293	**C_AA120870**	–	–	–	**C_AA121178**	Present study
*A. subfrostiana*	HKAS 58847	JN943172	JN941161	JQ031119	KR824806	MH485800	Schoch et al. 2012; Cui et al. 2018
*A. subfrostiana*	HKAS 76308	MH508616	MH486898	MH486300	–	–	Cui et al. 2018
*A. subfrostiana*	MHHNU 11320	**C_AA120871**	**C_AA120997**	**C_AA121026**	**C_AA121148**	**C_AA121179**	Present study
*A. subfrostiana*	MHHNU 32919	**C_AA120872**	**C_AA120998**	**C_AA121027**	**C_AA121149**	**C_AA121180**	Present study
*A. subfrostiana*	MHHNU 32955	**C_AA120873**	**C_AA120999**	**C_AA121028**	**C_AA121150**	**C_AA121181**	Present study
*A. subglobosa*	MHHNU 11257	**C_AA120874**	**C_AA121000**	**C_AA121029**	**C_AA121151**	**C_AA121182**	Present study
*A. subglobosa*	MHHNU 31083	MK388157	ON139048	ON229644	ON229684	ON125330	Su et al. 2023
*A. subglobosa*	MHHNU 31084	MK239245	ON139049	ON229645	ON229685	ON125331	Su et al. 2023
*A. subglobosa*	MHHNU 31308	ON131752	ON139050	ON229646	ON229686	ON125332	Su et al. 2023
*A. subglobosa*	MHHNU 32538	ON131753	ON139051	ON229647	ON229687	ON125333	Su et al. 2023
*A. subparcivolvata*	MHHNU 31557	OM955212	OM955208	OM949815	–	OM949822	Su et al. 2022
*A. subparcivolvata*	MHHNU 32368	OM955213	OM955211	OM949816	–	OM949823	Su et al. 2022
*A. subparcivolvata*	MHHNU 32849	OM955210	OM955209	OM949817	–	OM949824	Su et al. 2022
*A. subparcivolvata*	MHHNU 32907	OM955216	OM955205	OM949818	–	OM949825	Su et al. 2022
*A. subparcivolvata*	MHHNU 33170	OM955215	OM955214	OM949820	–	OM949827	Su et al. 2022
*A. subparvipantherina*	MHHNU 11319	**C_AA120875**	**C_AA121001**	**C_AA121030**	**C_AA121152**	**C_AA121183**	Present study
*A. subparvipantherina*	MHHNU 32163	ON131754	ON139051	ON229648	ON229688	ON125334	Su et al. 2023
*A. subparvipantherina*	MHHNU 32226	ON131756	ON139054	ON229650	ON229690	ON125336	Su et al. 2023
*A. subparvipantherina*	MHHNU 32354	ON131758	ON139056	ON229652	ON229691	ON125338	Su et al. 2023
*A. subparvipantherina*	MHHNU 32922	**C_AA120876**	**C_AA121002**	**C_AA121031**	**C_AA121153**	**C_AA121184**	Present study
*A. sychnopyramis*	MHHNU 32153	ON131761	ON139060	ON229654	ON229692	ON125342	Su et al. 2023
*A. sychnopyramis*	MHHNU 32799	ON131762	ON139061	ON229655	ON229660	ON125343	Su et al. 2023
*A. sychnopyramis*	MHHNU 33272	**C_AA120878**	**C_AA121004**	**C_AA121033**	**C_AA121155**	**C_AA121186**	Present study
*A. sychnopyramis*	MHHNU 33330	**C_AA120879**	**C_AA121005**	**C_AA121034**	**C_AA121156**	**C_AA121187**	Present study
*A. sychnopyramis*	MHHNU 33350	**C_AA120880**	**C_AA121006**	**C_AA121035**	**C_AA121157**	**C_AA121188**	Present study
*A. avellaneifolia*	HKAS 80011	–	MH486378	MH485872	MH508680	MH485410	Cui et al. 2018
*A. flavipes*	HKAS 88080	MH508346	MH486509	MH485981	MH508785	MH485505	Cui et al. 2018

The newly generated sequences submitted to GenBase are highlighted in bold.

### ﻿DNA extraction, PCR amplification, Sanger sequencing, and species identification

For Sanger sequencing samples, the Fungal DNA Mini Kit (Omega Bio-Tek, Norcross, USA) was used to extract total genomic DNA according to the manufacturer’s instructions. The following primers were employed for PCR amplification: (i) ITS5/ITS4 (White et al. 1990) for the internal transcribed spacer (ITS), (ii) LR0R/LR5 (Vilgalys and Hester 1990) for the nuclear ribosomal large subunit (LSU), (iii) Am-6F/Am-7R (Cai et al. 2014) for the DNA-directed RNA polymerase II subunit 2 (*RPB2*), (iv) EF1-983F/EF1-1567R (Rehner and Buckley 2005) for the translation elongation factor 1-α (*TEF1*), and (v) Am-β-tubulin F/Am-β-tubulin R (Cai et al. 2014) for beta-tubulin (*TUB2*).

The PCR mixture consisted of 1× PCR buffer, 1.5 mM MgCl_2_, 0.2 mM dNTPs, 0.4 μM forward primer, 0.4 μM reverse primer, 1.25 U of *Taq* polymerase (Comwin Biotech, Beijing, China), and 1 μL of DNA template in a total volume of 25 μL. Reactions were performed using the following program: initial denaturation at 94°C for 4 min, followed by 35 cycles at 94°C for 30 s, 54°C for 30 s, and 72°C for 30 s (ITS, *TEF1*, and *TUB2*) or 60 s (LSU and *RPB2*), with a final extension at 72°C for 2 min. Amplification products were examined by electrophoresis on a 1.5% agarose gel and sent to the Changsha branch of Tsingke Biological Technology Co., Ltd. (Beijing, China) for sequencing.

Using the online program BLAST (https://blast.ncbi.nlm.nih.gov/Blast.cgi), specimens were identified to species or genus level by using their sequences as queries. The resulting species names were further validated by comparing morphological data with descriptions presented in Cui et al. (2018). The taxon names used in this study follow MycoBank (https://www.mycobank.org; Robert et al. 2013), with authorities omitted.

### ﻿Genomic library preparation and NGS sequencing

For NGS samples, a modified cetyltrimethylammonium bromide (CTAB) protocol introduced by Li et al. (2013) was used for genomic DNA extraction. DNA concentration was determined using a Qubit 2.0 Fluorometer (Life Technologies, Carlsbad, CA, USA). DNA purity was assessed using a NanoPhotometer N80 (Implen, Munich, Germany), and DNA integrity was evaluated by electrophoresis on a 1% agarose gel. A total of 25 DNA samples representing different *Amanita* species (Suppl. material 1: table S1) were qualified for library construction. Paired-end (PE) libraries (insert size: 350 bp) were generated using the TruSeq Nano DNA HT Sample Preparation Kit (Illumina, San Diego, CA, USA) following the manufacturer’s protocol. NGS was performed on an Illumina NovaSeq 6000 platform (Illumina, San Diego, CA, USA) at Grandomics Biosciences Co., Ltd. (Wuhan, China). The sequencing data generated for each sample amounted to 5 Gbase.

### ﻿Data filtering and genome assembly

FastQC v0.11.9 (Andrews 2010) was used to assess raw data quality. Trimmomatic v0.36 (Bolger et al. 2014) was applied to generate clean data using the following parameters: ILLUMINACLIP: adapters.fa:2:30:10; LEADING:3; TRAILING:3; SLIDINGWINDOW:4:15; MINLEN:20. Clean data were evaluated using FastQC and SOAPec v2.03 (k-mer = 17; Luo et al. 2012) and subsequently used as input for de novo assembly using A5-miseq v[08/25/2016] (Coil et al. 2014) and SPAdes v3.13.1 (Prjibelski et al. 2020). Pilon v1.18 (Walker et al. 2014) was employed for assembly correction. Assembly-stats v1.0.1 (https://pypi.org/project/assembly-stats/) was used to summarize basic assembly information, and BUSCO v5.2.2 (Manni et al. 2021) was used to assess assembly completeness. Assembly statistics for 25 *Amanita* species are summarized in Suppl. material 1: table S1.

### ﻿Single-copy gene extraction and phylogenomic analysis

Red v[05/22/2015] (Girgis 2015) was used to filter repeated sequences. Augustus v3.3.3 (Keller et al. 2011), GlimmerHMM v3.0.1 (Majoros et al. 2004), GeneMark-ES v4.35 (Ter-Hovhannisyan et al. 2008), and Exonerate v2.2 (Slater and Birney 2005) were used for gene prediction. Predicted genes were integrated using EVidenceModeler v[06/25/2012] (Haas et al. 2008). Predicted proteins were extracted using gffread v0.12.7 (Pertea and Pertea 2020). Using BUSCO analysis, single-copy genes from 25 samples (Suppl. material 1: table S1) were retrieved by comparing predicted proteins against the Agaricales_odb10 lineage dataset (https://busco-data.ezlab.org/v4/data/lineages/). A total of 467 shared single-copy genes (Suppl. material 1: table S2) were selected for phylogenomic analysis. MAFFT v7.511 (Katoh and Standley 2016) and Gblocks v0.91b (Castresana 2000) were used for amino acid sequence alignment and conserved region extraction, respectively. The shared single-copy gene sequences were concatenated to form Dataset I (292,634 positions, Suppl. material 2). Maximum likelihood (ML) analysis with 1,000 bootstrap replicates was performed using FastTree v2.1.11 (Price et al. 2010) under the LG+CAT model, following Wang et al. (2019).

### ﻿*Ibo* gene mining and identification

Using the public protein sequences of the seven *ibo* genes from *A. muscaria* Koide BX008 (IboA: KIL56732, IboC: KIL56737, IboD: KIL56734, IboF: KIL56733, IboG1: KIL56738, IboG2: KIL56740, and IboH: KIL56739) as queries, BLAST+ v2.16.0 (Camacho et al. 2009) was employed with default parameters to search for translated nucleotide sequences of homologous genes and to retrieve the open reading frames (ORFs) of homologs from the 25 assembled genomes described above. The extracted candidate gene sequences were simultaneously aligned with the gene sequences and coding sequences (CDS) of the *ibo*BGC genes from *A. muscaria* to predict the CDS of *ibo* genes from other *Amanita* species. The resulting CDS were translated into amino acid sequences, and pairwise identity values with the corresponding *ibo*BGC proteins of *A. muscaria* were calculated using DNAMAN v7.212 (Lynnon Biosoft, Quebec, Canada) for identification.

### ﻿Phylogenetic analysis

To construct species trees, sequences of two loci (Table 2) and five loci (Table 3) were separately aligned using MAFFT. Ambiguously aligned regions of ITS and LSU were removed using Gblocks, and intron regions of *TEF1* and *RPB2* were manually excised. A concatenated ITS–LSU matrix (Dataset II, Suppl. material 2) and a concatenated ITS–LSU–*RPB2*–*TEF1*–*TUB2* matrix (Dataset III, Suppl. material 2) were generated using SequenceMatrix v1.7.8 (Vaidya et al. 2011). The two-locus Dataset II for the genus *Amanita* comprised 1475 positions from 184 samples, covering a worldwide sampling of 75 species from sect. Amanita and 19 representative species from the remaining 10 sections. The five-locus Dataset III for section Amanita consisted of 2888 positions from 101 samples representing 29 sect. Amanita species and two outgroup species, *A. avellaneifolia* (sect. Roanokenses) and *A. flavipes* (sect. Validae).

According to the AIC criterion in MrModeltest v2.4 (Nylander 2004), GTR+I+G was selected as the best-fit model for the ITS and LSU partitions in Datasets II and III, as well as for the *TEF1* partition in Dataset III. For the *RPB2* partition, SYM+I+G was selected, whereas for the *TUB2* partition, K80+I+G was chosen. Bayesian inference (BI) analysis was performed in MrBayes v3.2.7 (Ronquist and Huelsenbeck 2003) using the selected models, two simultaneous runs, four Markov chain Monte Carlo (MCMC) chains, sampling every 1000 generations, and a total of four million generations, ensuring that the standard deviation of split frequencies fell below 0.01. The first 25% of sampled generations were discarded as burn-in, and convergence of runs was visually assessed using the trace function in Tracer v1.7.2 (Rambaut et al. 2018). Maximum likelihood (ML) analysis with 1,000 bootstrap replicates was performed in RAxML v8.0.20 (Stamatakis 2014) using the GTR+G+I model for the entire dataset. Because the SYM+I+G and K80+I+G models are not implemented in RAxML, the GTR+I+G model was applied for ML analysis instead.

To construct gene trees, seven CDS matrices for each *ibo* gene (Datasets IV–X, Suppl. material 2) were compiled and analyzed individually following the procedure described above. In BI analyses, the models GTR+G (*iboA* and *iboG1*), HKY+G (*iboC*), SYM+G (*iboD*), GTR+I+G (*iboG2*), and SYM+I+G (*iboH*) were applied. In ML analyses, because RAxML does not support HKY+G, SYM+G, and SYM+I+G models, these were replaced by GTR+G, GTR+G, and GTR+I+G, respectively.

### ﻿Divergence time estimation

A two-step calibration method was used to estimate node ages in BEAST v1.10.4 (Suchard et al. 2018), following Zhao et al. (2016). Nodes representing *Ganoderma*, *Hymenochaetales*, *Suillaceae*, and the marasmioid clade were selected for calibration based on Varga et al. (2019). Dataset XI (2892 positions, Suppl. material 2) comprised ITS, LSU, *RPB2*, and *TEF1* sequences from 81 representatives of *Agaricomycetes* (Suppl. material 1: table S3) and was used to incorporate calibration points. Introns within *RPB2* and *TEF1*, as well as ambiguously aligned regions of ITS and LSU, were removed. The XML file was generated in BEAUti v1.10.4 with the following settings: GTR+G+I substitution model for each partition, an uncorrelated lognormal relaxed clock (Drummond et al. 2006), a Yule process tree prior (Gernhard 2008), 200 million generations, and sampling every 1000 generations. Output log files from two independent runs were examined in Tracer to assess convergence and confirm effective sample sizes (ESS) ≥ 200. After discarding the first 10% of sampled generations as burn-in, the maximum clade credibility (MCC) tree was generated using TreeAnnotator v1.10.4.

Dataset XII (2597 positions, Suppl. material 2) comprised ITS, LSU, *RPB2*, and *TEF1* sequences from 145 samples representing 73 species of *Amanitaceae* and two species of *Pluteaceae* (Suppl. material 1: table S4). This dataset was analyzed following a procedure similar to that applied to Dataset XI. The split between *Amanitaceae* and *Pluteaceae*, as estimated from Dataset XI, was used as the calibration point. A normal distribution prior with a standard deviation of 1.0 was applied to the treeModel.rootHeight parameter. Node ages were calibrated using the highest posterior density (HPD) age values.

### ﻿Toxin extraction and detection

Toxin extraction and detection were carried out according to the methods described by Li et al. (2022b) and Zhang et al. (2022). A total of 101 dried mushroom samples (Table 1; 10 mg per sample) were individually ground into a fine powder. The powder was mixed with 2 mL of methanol–deionized H_2_O (7:3, v/v) using an ultrasonic bath for 60 min, followed by centrifugation at 250 g for 5 min at 4°C. The supernatant was purified using a QuCHERS-PP column. A 10 μL aliquot of the purified extract was diluted with methanol–deionized H_2_O (5:95, v/v) to a final volume of 1.0 mL. The resulting solution was centrifuged at 40,000 g for 2 min. Supernatants from each sample were diluted fortyfold or two-hundredfold for subsequent detection by ultrahigh-performance liquid chromatography–tandem mass spectrometry (UHPLC–MS/MS) analysis. *Lentinula edodes* was used as a blank control.

UHPLC–MS/MS analysis was performed using a Waters ACQUITY I-Class UPLC system coupled with a Waters Xevo TQ-S MS/MS system (Waters, Milford, MA, USA), with chromatographic separation achieved using an ACQUITY UPLC BEH Amide column (100 mm × 2.1 mm, 1.7 μm; Waters). Mobile phase A consisted of 10 mmol/L ammonium acetate–0.05% formic acid aqueous solution, and mobile phase B was acetonitrile, with a flow rate of 0.3 mL/min. The gradient elution program was as follows: 0.1–2 min, 5% B; 2–3.5 min, 5–80% B; 3.5–4.5 min, 80% B; 4.5–5 min, 80–5% B; and 5–7 min, 5% B. The column temperature was maintained at 40°C, and the injection volume was 10 μL. Mass spectrometric analysis was performed in positive electrospray ionization (ESI^+^) mode with the following parameters: capillary voltage, 3 kV; cone voltage, 18 V; collision voltage, 16 V; desolvation temperature, 500°C; desolvation gas flow, 1000 L/h; cone gas flow, 150 L/h; collision gas flow, 11.4 L/h; all other parameters were set to default values. In multiple reaction monitoring mode, the protonated molecular ion ([M+H]^+^) at m/z 174.2 was selected as the parent ion, and two product ions at m/z 57.1 and 97.2 were used for quantitative and qualitative detection, respectively.

Muscarine standard (C_9_H_20_NO_2_.Cl, 209.7; CAS No. 2303-35-7; 98% purity) was purchased from Good Laboratory Practice Bioscience Co., Ltd. (Montclair, CA, USA). A calibration curve for quantitative analysis was constructed using a series of standard dilutions (0.1, 0.5, 1, 5, and 10 ng/mL). Muscarine was scored as present or absent by comparing sample chromatograms with that of the standard, and its content was expressed as mg/kg on a dry-weight basis.

## ﻿Results

### ﻿Barcode-based phylogenetic analysis

**Figure 2.**
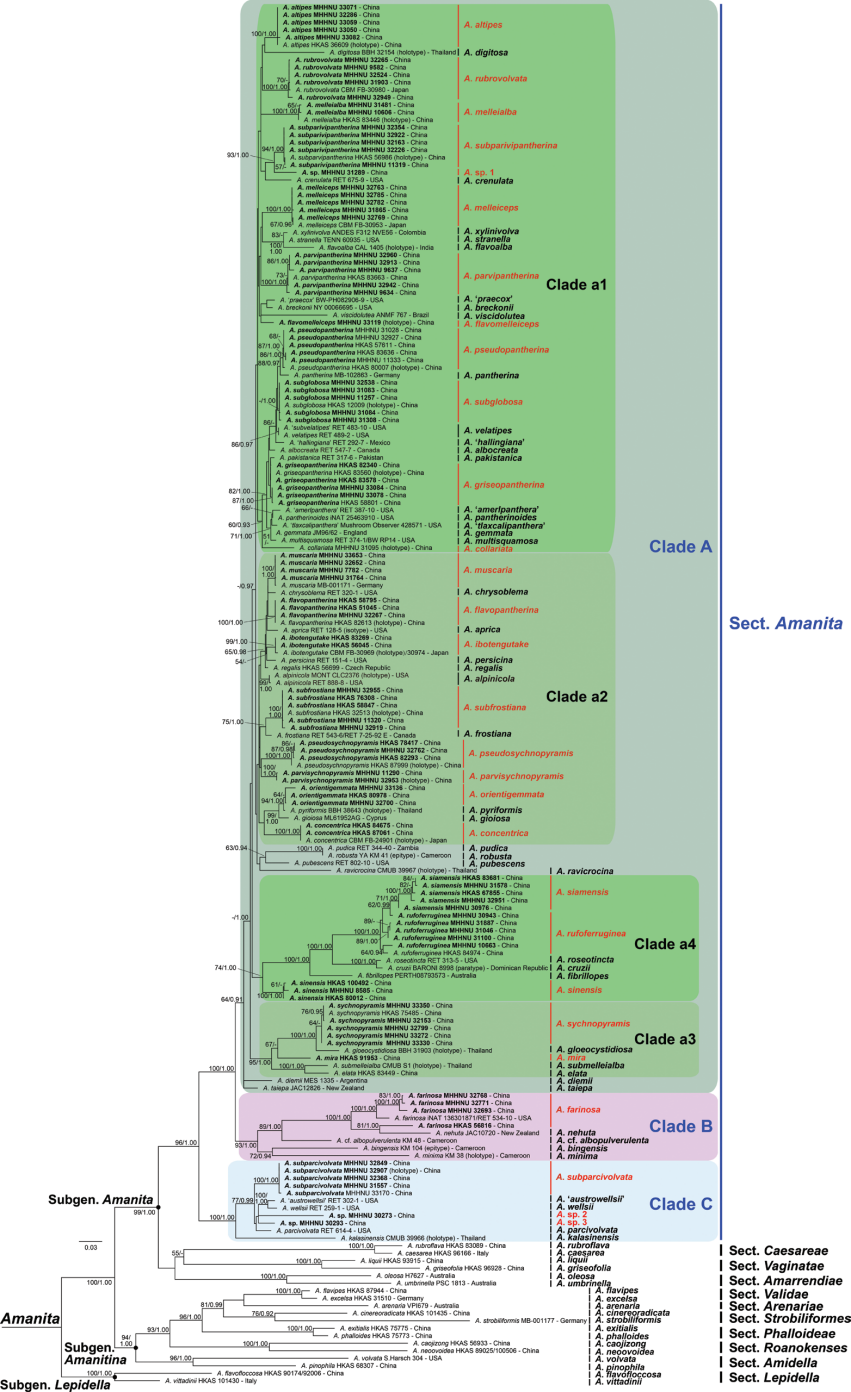
ML analysis of *Amanita* based on concatenated ITS–LSU sequence data from a worldwide sampling, with a focus on sect. Amanita, rooted with *A. flavofloccosa* and *A. vittadinii* (sect. Lepidella, subgen. Lepidella). Bootstrap values (BP) ≥ 50% from ML analysis and Bayesian posterior probabilities (PP) ≥ 0.90 from BI analysis are shown at nodes. Geographic origins of all specimens are presented, and type specimens are marked in parentheses. Chinese specimens used in this study are highlighted in bold, and their species names are indicated in red.

As shown in Fig. 2, a phylogenetic tree of Amanita emphasizing sect. Amanita was constructed. Using the commonly applied barcodes ITS and LSU, the analysis covered 75 species of sect. Amanita worldwide and recovered the section as three distinct clades: Clade A (64% BP, 0.91 PP), Clade B (93% BP, 1.00 PP), and Clade C (100% BP, 1.00 PP). The robust Clade A comprised four major subclades: Clades a1 and a2 (unsupported), a3 (95% BP, 1.00 PP), and a4 (74% BP, 1.00 PP). The analysis further revealed that the 99 Chinese samples represented 29 species belonging to sect. Amanita, including 26 previously described species and three unnamed species, distributed across Clades a1–a4, B, and C.

**Figure 3.**
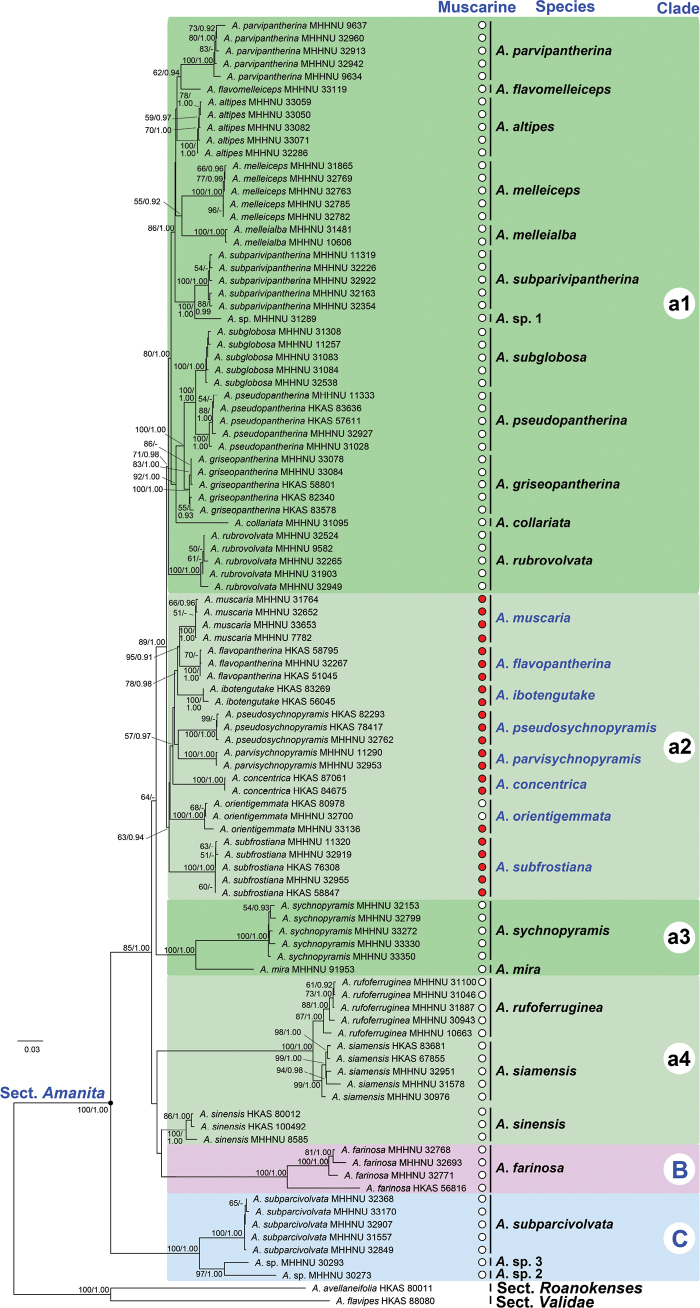
ML analysis of Amanita sect. Amanita based on concatenated ITS–LSU–*RPB2*–*TEF1*–*TUB2* sequence data, with *A. avellaneifolia* (sect. Roanokenses) and *A. flavipes* (sect. Validae) as outgroups. Bootstrap values (BP) ≥ 50% from ML analysis and Bayesian posterior probabilities (PP) ≥ 0.90 from BI analysis are shown at nodes. Red and white circles represent the presence and absence of muscarine, respectively, and muscarine-containing species are highlighted in blue.

To verify the subdivision of these clades, a five-locus phylogenetic tree of sect. Amanita (Fig. 3), encompassing the 29 representative species, was constructed. In this analysis, Clades B (100% BP, 1.00 PP) and C (100% BP, 1.00 PP) were confirmed with full support. Clades a1 (80% BP, 1.00 PP) and a3 (100% BP, 1.00 PP) received strong to full support, whereas Clade a2 (64% BP, 0.94 PP) received weak ML support but significant BI support. Clade a4 was divided into two subclades that did not cluster together, and its topological structure lacked statistical support.

### ﻿Phylogenomic analysis

**Figure 4.**
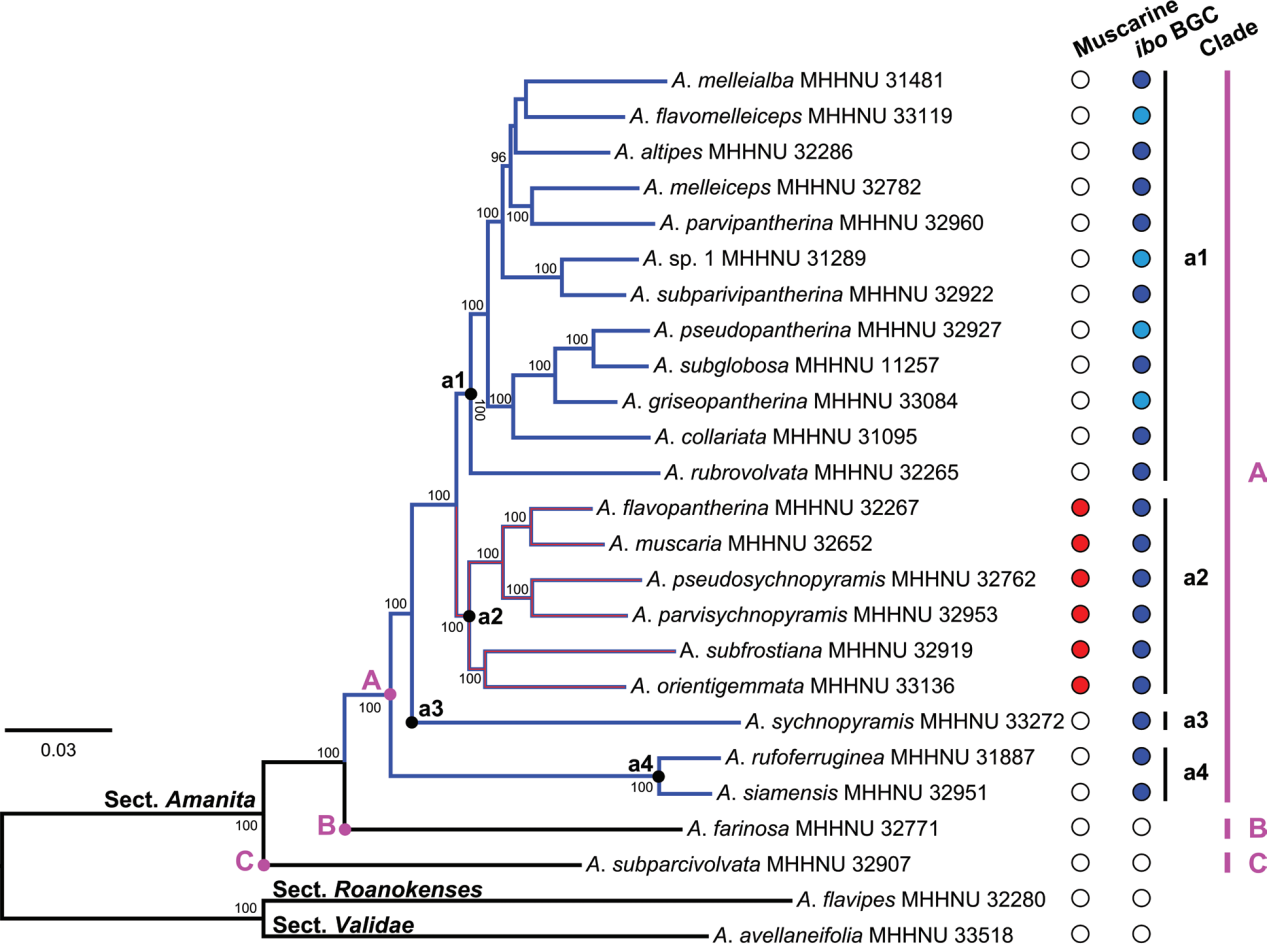
ML analysis of sect. Amanita based on 467 single-copy genes showing the major clades within the section. Red and dark blue circles indicate the presence of muscarine and the entire *ibo*BGC, respectively, and white circles indicate absence. Sky blue indicates that the *ibo*BGC was not fully recovered from the available genome data. *A. flavomelleiceps*: *iboG1*, *iboG2*, and *iboH* were not obtained; *A. griseopantherina*: *iboF* was not obtained; *A. pseudopantherina*: *iboC*, *iboG2*, and *iboH* were not obtained; *Amanita* sp. 1: only *iboA* was obtained.

To completely resolve the internal clades within the framework of sect. Amanita, a phylogenomic tree of the section, was constructed based on 467 single-copy genes from 25 species, including 23 representative species from sect. Amanita and two outgroup species from sects. *Roanokenses* and *Validae*. Most nodes within the sect. Amanita that lacked support or showed weak support in the two multilocus trees (Figs 2, 3) were strongly supported in the 467-gene tree (Fig. 4), resulting in a well-resolved topology. According to the phylogenomic analysis, Clades A–C and subclades a1–a4 within the species-rich Clade A were all supported with 100% bootstrap values. This resolved the previously ambiguous arrangement of the four subclades a1–a4 in the phylogenetic analyses.

### ﻿Muscarine detection

A total of 101 samples from sects. *Amanita* (99), *Roanokenses* (1), and *Validae* (1) were analyzed using UHPLC–MS/MS. The sect. Amanita samples represented 29 species distributed among different clades: Clades a1 (12), a2 (8), a3 (2), a4 (3), B (1), and C (3). Muscarine was detected in 22 samples from eight sect. Amanita species: *A. concentrica*, *A. flavopantherina*, *A. ibotengutake*, *A. muscaria*, *A. orientigemmata*, *A. pseudosychnopyramis*, *A. parvisychnopyramis*, and *A. subfrostiana*. The concentrations detected in the dry basidiomata of these species ranged from 1.09 to 6428.57 mg/kg. Detailed detection results for each sample are provided in Table 1.

### ﻿Identification of *ibo* BGC genes

Using the seven known *ibo*BGC genes from *A. muscaria* Koide BX008 (Obermaier and Müller 2020) as tblastn queries, 134 *ibo* genes (21 *iboA*, 19 *iboC*, 20 *iboD*, 19 *iboF*, 19 *iboG1*, 18 *iboG2*, and 18 *iboH*; Suppl. material 1: table S5) were identified from the genomes of 25 *Amanita* species. The ibo genes were present in 21 species from sect. Amanita but absent in *A. farinosa* (sect. Amanita), *A. subparcivolvata* (sect. Amanita), *A. avellaneifolia* (sect. Roanokenses), and *A. flavipes* (sect. Validae). All seven *ibo*BGC genes were successfully retrieved from 17 species, whereas only a subset of these seven *ibo* genes was obtained from each of the remaining four species, most likely owing to DNA degradation. Each *ibo* gene from *A. muscaria*MHHNU 32652 exhibited 100% amino acid identity with those of *A. muscaria* Koide BX008. The *ibo* genes of the other 20 species showed amino acid identities ranging from 70.02% to 97.09% compared with those of *A. muscaria* Koide BX008, with most values exceeding 80% (Suppl. material 1: table S5).

### ﻿Phylogenetic distribution of muscarine and the *ibo* BGC

The results of the species tree analyses and muscarine detection are summarized in Fig. 3. The five-locus phylogram indicates that all muscarine-containing species within the sect. Amanita forms a monophyletic clade (Clade a3), although this clade is supported by weak ML support (63% BP). However, this monophyletic grouping is fully supported (100% BP) in the 467-gene phylogram shown in Fig. 4, further confirming the monophyletic distribution of muscarine-containing species.

The distribution of the *ibo*BGC within the sect. Amanita is shown in Fig. 4. As illustrated, the 21 species possessing *ibo* genes cluster together to form a robust monophyletic clade, viz. Clade A, with full ML support (100% BP). The species *A. farinosa* and *A. subparcivolvata*, representing Clades B and C, respectively, and lacking the *ibo*BGC, occupy subbasal and basal positions within the phylogeny of sect. Amanita.

### ﻿*Ibo* gene-based phylogenetic analysis

**Figure 5.**
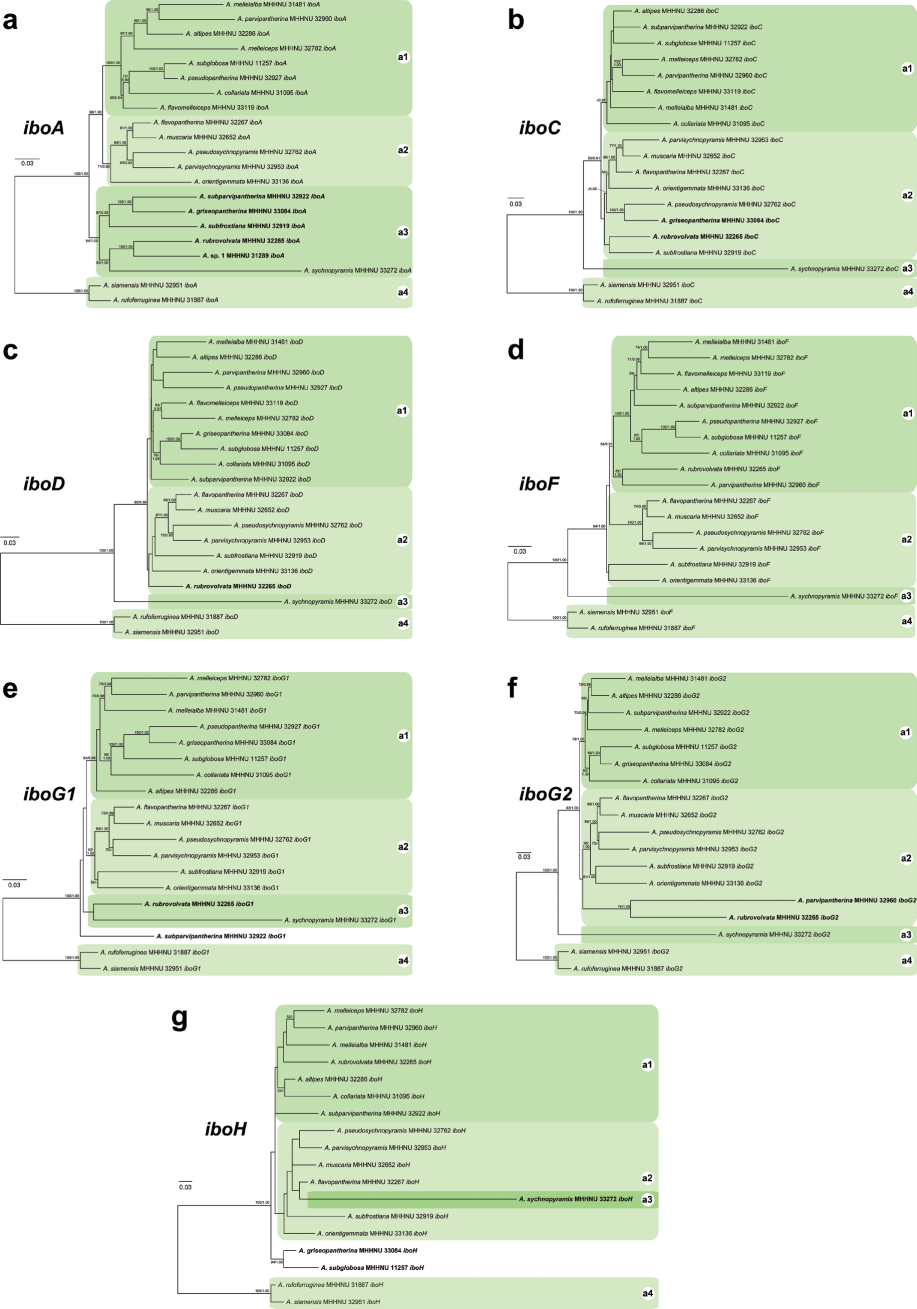
ML analysis of Clade A within the sect. Amanita based on the coding sequences of *iboA* (**a**), *iboC* (**b**), *iboD* (**c**), *iboF* (**d**), *iboG1* (**e**), *iboG2* (**f**), and *iboH* (**g**), respectively. Bootstrap values (BP) ≥ 50% from ML analysis and Bayesian posterior probabilities (PP) ≥ 0.90 from BI analysis are shown at nodes. Genes that are in conflict with the organismal phylogenetic clades are highlighted in bold.

To investigate the evolution of the *ibo*BGC, phylogenetic analyses were conducted for each individual gene (Fig. 5). The phylogenetic relationships inferred for these seven genes were not entirely congruent with the organismal phylogeny in terms of topology. Each *ibo* gene (Fig. 5a–g) was recovered as four clades (Clades a1–a4), consistent with the phylogenetic clade divisions of the corresponding species (Figs 2–4). For *iboF* (Fig. 5d), the gene tree was largely topologically consistent with the species tree (Fig. 4). However, in the other gene trees, the *iboA* and *iboG1* genes of *A. rubrovolvata*, a species belonging to Clade a1 in the species tree, were instead placed in Clade a3 (Fig. 5a, e), whereas its *iboC*, *iboD*, and *iboG2* genes were placed in Clade a2 (Fig. 5b, c, f). The *iboA* gene of *A. griseopantherina* (species tree: a1) was placed in Clade a3 (Fig. 5a), whereas its *iboC* gene was placed in Clade a2 (Fig. 5b). The *iboA* genes of *Amanita* sp. 1 (a1), *A. subfrostiana* (a2), and *A. subparvipantherina* (a1) were placed in Clade a3 (Fig. 5a), whereas the *iboG1* gene of *A. subparvipantherina* formed a distinct clade separate from Clades a1–a4 (Fig. 5e). The *iboG2* gene of *A. parvipantherina* (a1) was placed in Clade a2 (Fig. 5f). The *iboH* genes of *A. griseopantherina* and *A. subglobosa* (a1) deviated from their species clades, whereas the *iboH* gene of *A. sychnopyramis* (a3) was nested within Clade a2 (Fig. 5g).

### ﻿Molecular dating

**Figure 6.**
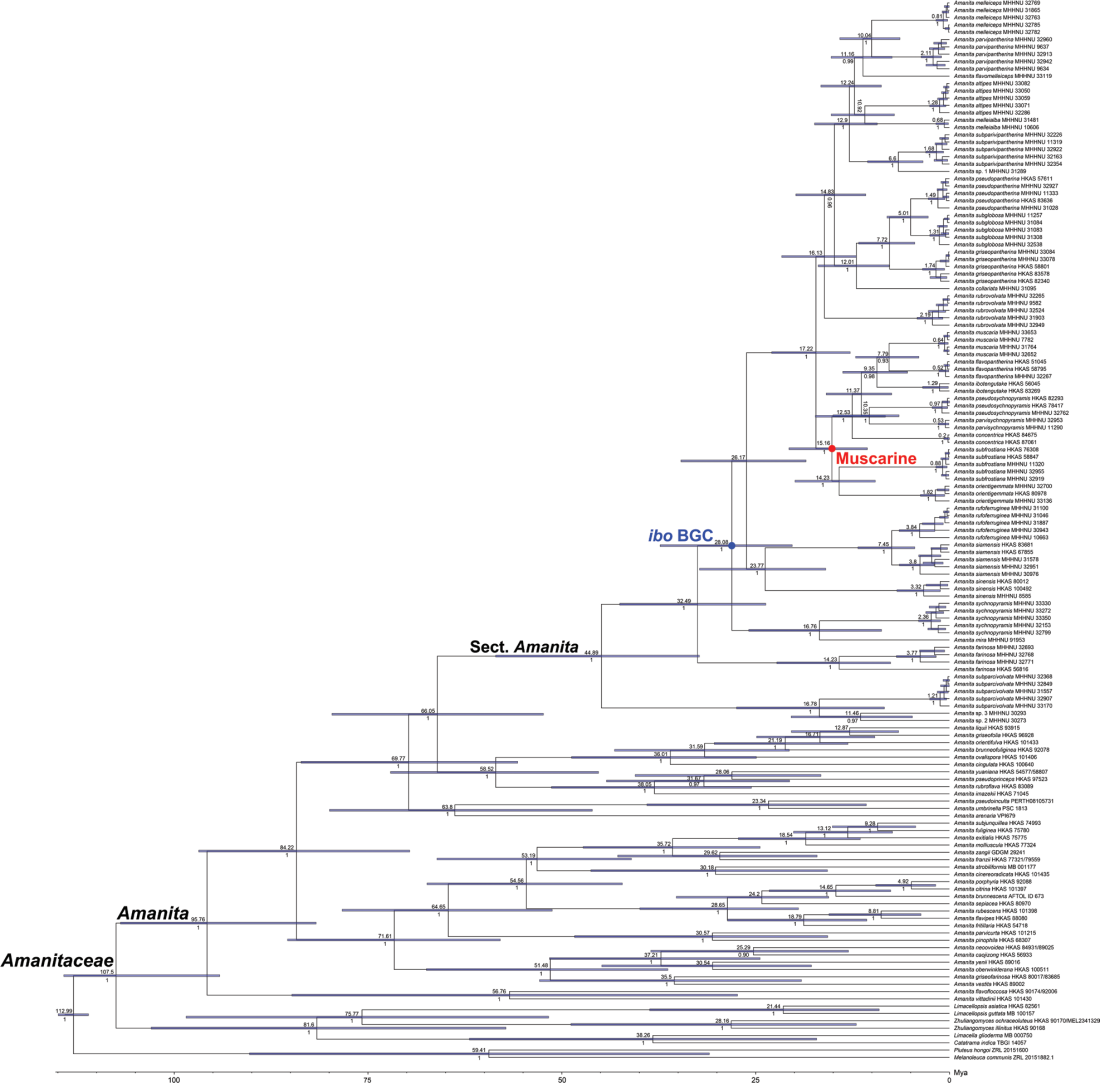
MCC tree of *Amanitaceae* based on concatenated ITS–LSU–*RPB2*–*TEF1* sequence data, with *Melanoleuca communis* and *Pluteus hongoi* (*Pluteaceae*) as outgroups. The estimated median divergence times and Bayesian posterior probabilities ≥ 0.90 are shown above and below each node, respectively. The 95% highest posterior density of divergence times is indicated as blue node bars.

The MCC tree of *Agaricomycetes* (Suppl. material 3: fig. S1) indicated that the split between *Amanitaceae* and *Pluteaceae* occurred during the Cretaceous period, with a median estimate of 113.03 Mya (95% HPD: 97.09–130.07 Mya), consistent with the estimate reported by Varga et al. (2019). Using this divergence time as the calibration point, the estimated divergence times of *Amanitaceae*, *Amanita*, and sect. Amanita (Fig. 6) were 107.50 Mya (94.21–114.40 Mya), 95.76 Mya (81.53–106.96 Mya), and 44.89 Mya (32.92–57.20 Mya), respectively. Within the sect. Amanita, the acquisition of muscarine and the *ibo*BGC was estimated to have occurred at 15.16 Mya (10.66–20.02 Mya) and 28.08 Mya (20.28–37.32 Mya), respectively.

## ﻿Discussion

### ﻿Taxonomic distribution and evolution of the ibo BGC within the section Amanita

Analyses aiming to resolve the relationship between phylogeny and the presence of the *ibo*BGC within the sect. Amanita has not been performed previously. Based on our phylogenetic framework of sect. Amanita (Figs 2–4), we provide a comprehensive and representative sampling of species within the section that were assayed for the *ibo*BGC. Although the 23 species analyzed are all from China (Fig. 4), they represent almost all clades of the ITS tree inferred from publicly available sequences of sect. Amanita (Fig. 2). According to our phylogenetic and genomic findings (Fig. 4), the *ibo*BGC is present exclusively in species belonging to Clade A of sect. Amanita and is absent from species in the other two clades, Clades B and C. Furthermore, the taxonomic distribution of the *ibo*BGC is monophyletic, providing a more accurate and clearer elucidation of the correlation between phylogeny and the production of ibotenic acid and muscimol than our previous findings reported in Su et al. (2023).

The monophyletic distribution of the *ibo*BGC within the sect. Amanita can also be corroborated by previous studies, including BGC analyses, toxin detection efforts, and reports of poisoning incidents involving species from this section that present symptoms associated with ibotenic acid and muscimol exposure. As shown in Fig. 2, the North American species *A. aprica*, which has caused similar poisonings (Tulloss and Lindgren 2005), belongs to Clade A of sect. Amanita. The European and Japanese ibotenic acid and muscimol-containing species, *A. gemmata*, *A. regalis*, and *A. ibotengutake* (Benedict et al. 1966; Oda et al. 2002), are also nested within Clade A. Obermaier and Müller (2020) first demonstrated three *ibo*BGC-containing species, *A. crenulata*, *A. muscaria*, and *A. pantherina*, all of which are distributed within Clade A, as expected.

In the present work, we identified *ibo* BGCs in 21 Chinese species belonging to Clade A of sect. Amanita (Fig. 4). Among these species, 10—viz. *A. altipes*, *A. flavopantherina*, *A. griseopantherina*, *A. muscaria*, *A. parvisychnopyramis*, *A. pseudopantherina*, *A. pseudosychnopyramis*, *A. rubrovolvata*, *A. subglobosa*, and *A. sychnopyramis*—were confirmed to produce ibotenic acid and muscimol by UHPLC–MS/MS in our previous studies (Su et al. 2023, 2025). These chemical analysis results are consistent with our current findings from *ibo*BGC gene identification. Eight additional species—viz. *A. collariata*, *A. melleialba*, *A. melleiceps*, *A. orientigemmata*, *A. parvipantherina*, *A. rufoferruginea*, *A. siamensis*, and *A. subfrostiana*—have been responsible for numerous neurotoxic poisoning incidents in China over the past 5 years (Li et al. 2021a, 2022a, 2023, 2024, 2025). These poisoning reports correlate with our finding that these species possess the *ibo*BGC. Regarding the remaining three species—viz. *A. flavomelleiceps*, *A. subparvipantherina*, and *Amanita* sp. 1—no ibotenic acid or muscimol was detected in our specimens (Su 2023; Su et al. 2025). Nevertheless, the presence of *ibo*BGC genes in these species indicates that they are capable of producing these toxins. This may be attributable to degradation of the toxins to levels below the detection limit or their transformation into other isoxazole derivatives following drying and long-term preservation. Additionally, Su et al. (2023) reported the absence of ibotenic acid and muscimol in *A. farinosa* and *A. subparcivolvata*. Consistent with these findings, our analysis confirms that both species lack the *ibo*BGC required for toxin production.

It should be noted that incomplete *ibo* BGCs were obtained for four species, viz. *A. flavomelleiceps*, *A. griseopantherina*, *A. pseudopantherina*, and *Amanita* sp. 1 (Fig. 4; Suppl. material 1: table S5). This outcome reflects incomplete genome assembly, a common issue when sequencing dried mushroom specimens, as also observed by Bradshaw et al. (2024). Owing to DNA degradation in dried specimens, the resulting genome assemblies are highly fragmented (Suppl. material 1: table S1). For example, in *A. griseopantherina*, six genes (*iboA*, *iboC*, *iboD*, *iboG1*, *iboG2*, and *iboH*) were identified within the BGC, whereas *iboF* was not detected. Nevertheless, *A. griseopantherina* was previously shown to produce muscimol (Su et al. 2023). In the absence of *iboF*, muscimol biosynthesis would not be possible, indicating that this species should possess the complete *ibo*BGC. Therefore, the non-detection of certain *ibo* genes in these species is more likely an analytical artifact than a true biological absence.

The phylogenetic distribution pattern of *ibo*BGC genes within A. sect. Amanita closely resembles that of amanitin biosynthetic genes within A. sect. Phalloideae. In both cases, these genes are consistently detected in all species within each section, with the exception of the earliest or relatively early-diverging species (Hallen et al. 2007; Cai et al. 2014, 2016; Pulman et al. 2016; He et al. 2020). As shown in Figs 2–4, the species diversity of Clade A within the sect. Amanita in China, and globally, is evidently much greater than that of Clades B and C. The capacity to produce ibotenic acid and muscimol might be a contributing factor in accelerating or promoting speciation.

By comparing gene trees with species trees, Bradshaw et al. (2024) reported that phylogenies of psilocybin BGC genes, viz. *psiD*, *psiM*, *psiK*, and *psiH*, were largely to completely congruent with the species phylogeny of *Psilocybe*. In contrast, we found that topological consistency between *ibo*BGC gene trees and the species tree in sect. Amanita (Figs 4, 5) is lower. As shown in Fig. 5, the incongruence lies in differences in species composition across clades and in topological inconsistencies within clades. These inconsistencies may result from gene recombination, duplication, and loss, among other factors, and warrant further investigation. Similar gene tree–species tree conflicts have been reported for amanitin-associated genes, viz. MSDIN, POPB, FMO1, and P450-29, within A. sect. Phalloideae (Luo et al. 2018, 2022; He et al. 2020).

### ﻿Taxonomic distribution and evolution of muscarine within the section Amanita

To our knowledge, *A. muscaria* and *A. pseudosychnopyramis* were previously the only two species in sect. Amanita confirmed to contain muscarine (Eugster and Schleusener 1969; Chen et al. 2022). Our study extends the investigation of muscarine production to additional related species within this section. Besides these two species, we demonstrate for the first time that *A. concentrica*, *A. flavopantherina*, *A. ibotengutake*, *A. orientigemmata*, *A. parvisychnopyramis*, and *A. subfrostiana* contain muscarine. According to our phylogenetic and biochemical results (Figs 3, 4), these muscarine-containing species form a small monophyletic subclade within Clade A of sect. Amanita, whereas muscarine was not detected in species from other subclades of Clade A or from Clades B and C of the section.

The monophyletic distribution of muscarine in sect. Amanita is analogous to that observed in the family *Clitocybaceae*. In *Clitocybaceae*, muscarine represents an ancestral trait for a major clade, without subsequent losses. In sect. Amanita, it likewise represents an ancestral trait for a smaller clade, also with no subsequent losses. The acquisition of muscarine in sect. Amanita occurred approximately 15 Mya, slightly later than its emergence in *Clitocybaceae* at around 20 Mya (He et al. 2023). In contrast, the distribution of muscarine in *Inocybaceae* shows a weaker phylogenetic correlation, with muscarine present in five of its seven major clades, in which muscarine has evolved independently on several occasions, together with several losses (Kosentka et al. 2013).

### ﻿Toxicity and harmfulness of poisonous species of section Amanita

Our study reveals that a large clade of species within the sect. Amanita (Clade A, Figs 2–4) has the genetic capacity to produce ibotenic acid and muscimol. Ingestion of these poisonous mushrooms from sect. Amanita can lead to central nervous system excitation (White et al. 2019). Notably, we also found that a small subclade of species (Clade a2, Figs 2–4) within Clade A concurrently produces muscarine, resulting in additional parasympathetic nervous system toxicity (Peredy and Bradford 2014). In our biochemical study, muscarine was detected as positive in eight *Amanita* species from 22 samples. The concentrations of muscarine in dry basidiomata ranged from 1 to 6429 mg/kg (Table 1). The obtained muscarine concentration data for *Amanita* have significantly revised our previous understanding. Muscarine was previously considered to be present at a very low level in *A. muscaria*, with the content accounting for approximately 0.0003% of fresh weight (Puschner 2018). This has led to the historical neglect of muscarine in *Amanita* mushrooms.

In Chinese *A. muscaria*, muscarine was detected at concentrations ranging from 84 to 474 mg/kg, evidently higher than previously reported levels (Spoerke and Rumack 1994). In *Clitocybaceae*, *Clitocybe dealbata* has been reported to contain up to 1,800 mg/kg of muscarine (Genest et al. 1968). In *Inocybaceae*, *Inosperma muscarium* has been detected to contain 16,030 mg/kg of muscarine (Deng et al. 2021). In contrast, our results show that *A. concentrica* contains up to 6429 mg/kg of muscarine. The muscarine concentration in species of sect. Amanita can reach relatively high levels. The lethal dose of muscarine for humans is not precisely known, with estimates ranging from 180 to 300 mg (Bresinsky and Besl 1990) and from 40 to 495 mg (Benjamin 1995). Based on our concentration data, muscarine alone in certain species of sect. Amanita is sufficient to induce fatal poisoning, without the involvement of ibotenic acid and muscimol. Therefore, greater caution should be exercised regarding *Amanita* mushrooms containing the two types of toxin.

## ﻿Conclusion

Most species of Amanita sect. Amanita have the genetic capacity to produce ibotenic acid and muscimol, and these species collectively form a monophyletic clade. Within this clade, a subclade of species has the additional ability to produce muscarine. Within the sect. Amanita, the biosynthesis of ibotenic acid occurred earlier in evolutionary terms than that of muscarine, and both compounds appear to have originated once and been retained without subsequent losses. Overall, this study improves our understanding of toxicity profiles and the taxonomic distribution of toxins within A. sect. Amanita, thereby enabling healthcare providers to more effectively diagnose and treat affected individuals.

## ﻿Additional information

### Use of AI

No use of AI was reported.

### Author ORCIDs

Yu-Ting Su https://orcid.org/0009-0000-4194-5033

Zheng-Mi He https://orcid.org/0000-0001-8754-3427

Yu-Zhi Yang https://orcid.org/0009-0001-1908-7244

Fei Xu https://orcid.org/0000-0001-9219-1235

Meng-Meng Lai https://orcid.org/0009-0004-6519-6654

Zhu L. Yang https://orcid.org/0000-0001-9745-8453

Ping Zhang https://orcid.org/0000-0002-8751-704X

Zuo-Hong Chen https://orcid.org/0000-0001-7359-0257

### Data availability

The DNA sequences generated in this study have been submitted to GenBase (https://ngdc.cncb.ac.cn/genbase/). Alignments for phylogenetic, phylogenomic, and divergence time estimation analyses have been uploaded to TreeBASE under submission ID 32403. Raw short-read genomic sequences have been deposited in the Sequence Read Archive (SRA).

## Supplementary materials

Supplementary material 1 Supplementary tables S1–S5 Data type xlsx Explanation note **table S1**: Samples successfully sequenced using NGS and their corresponding summary statistics of genome assembly. The lineage dataset is *Agaricales*_odb10 with total 3870 BUSCO groups searched. **table S2**: The IDs of the 467 shared single-copy genes from 25 *Amanita* genomes within the *Agaricales*_odb10 dataset of BUSCO. **table S3**: Sequence used for estimating divergence time within *Agaricomycetes*. **table S4**: Sequence used for estimating divergence time within *Amanitaceae*. **table S5**: Amino acid sequence similarities of *ibo* genes between our Chinese species and *Amanita muscaria* Koide BX008, along with their GenBase accession numbers. https://binary.pensoft.net/file/1508557This dataset is made available under the Open Database License (http://opendatacommons.org/licenses/odbl/1.0/). The Open Database License (ODbL) is a license agreement intended to allow users to freely share, modify, and use this Dataset while maintaining this same freedom for others, provided that the original source and author(s) are credited.Zheng-Mi He

Supplementary material 2 Datasets I–XII Data type rar https://binary.pensoft.net/file/1508558This dataset is made available under the Open Database License (http://opendatacommons.org/licenses/odbl/1.0/). The Open Database License (ODbL) is a license agreement intended to allow users to freely share, modify, and use this Dataset while maintaining this same freedom for others, provided that the original source and author(s) are credited.Zheng-Mi He

Supplementary material 3 MCC tree of *Agaricomycetes* Data type pdf Explanation note **fig. S1**: MCC tree of *Agaricomycetes* based on concatenated ITS-LSU-*RPB2*-*TEF1* sequence data. The estimated median divergence times and Bayesian posterior probabilities ≥ 0.90 are above and below each node, respectively. https://binary.pensoft.net/file/1508559This dataset is made available under the Open Database License (http://opendatacommons.org/licenses/odbl/1.0/). The Open Database License (ODbL) is a license agreement intended to allow users to freely share, modify, and use this Dataset while maintaining this same freedom for others, provided that the original source and author(s) are credited.Zheng-Mi He
