# Supplementary material for: ﻿The distribution and evolution of muscarine and the ibotenic acid biosynthetic gene cluster within the genus Amanita section Amanita revealed by phylogenomics
Source: IMA Fungus. 2026 Jan 9;17:e175874. doi: 10.3897/imafungus.17.175874 (PMC12811753; doi:10.3897/imafungus.17.175874)
Supplement: Supplementary material 3 — MCC tree of Agaricomycetes [file imafungus-17-e175874-s003.pdf]

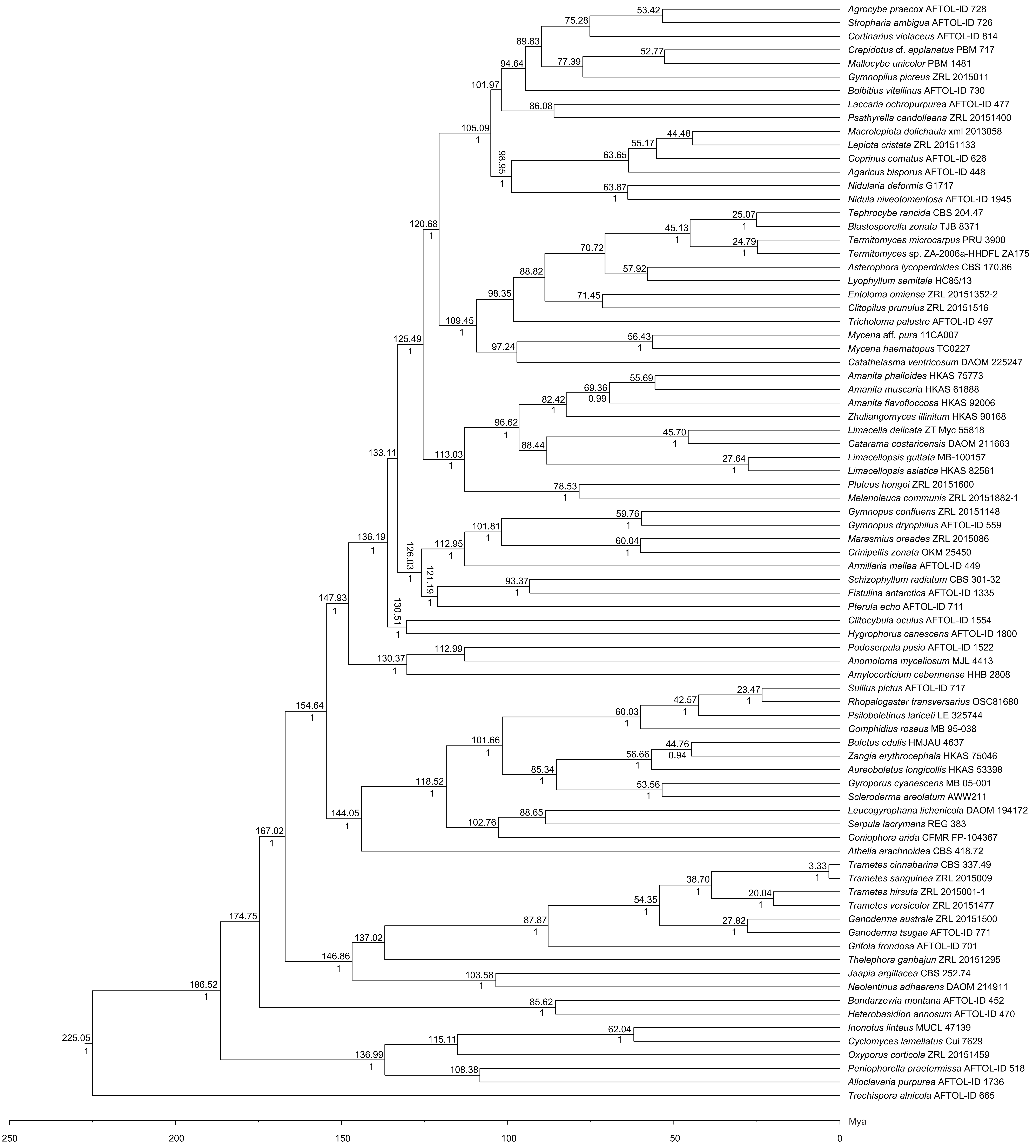

Fig. S1 MCC tree of *Agaricomycetes* based on concatenated ITS-LSU-*RPB2*-*TEF1* sequence data. The estimated median divergence times and Bayesian posterior probabilities ≥ 0.90 are above and below each node, respectively.
